# Comparative evaluation of air phytoremediation potential of four ornamental potted plants for ecofriendly biofilter applications

**DOI:** 10.1038/s41598-025-30145-8

**Published:** 2025-12-29

**Authors:** Safinaz M. Elhadad, E. A. Shalaby, I. H. Saleh, Mohamed Y. Omar

**Affiliations:** 1 Arab Academy for Science, Technology and Maritime Transport, Alexandria, Egypt; 2 Department of Environmental Studies, Institute of Graduate Studies and Research, Alexandria, Egypt; 3 Deanery of Scientific Research & Innovation, Arab Academy for Science, Technology and Maritime Transport, Alexandria, Egypt

**Keywords:** Air phytoremediation, Indoor air pollution, Pharmaceutical laboratory, VOC, PBBF, Potted plant, Environmental sciences, Plant sciences

## Abstract

Indoor air pollution poses serious health risks, especially in enclosed pharmaceutical laboratories due to volatile organic compounds (VOCs). This study evaluates Air Phytoremediation (AP) as a sustainable mitigation strategy using four indoor plant species: *Epipremnum aureum*, *Chlorophytum comosum*, *Syngonium podophyllum*, and *Cordyline fruticosa*. The removal efficiency of VOCs Benzene, Toluene, Benzaldehyde, and Acetophenone was assessed across various environments, including incubator chambers, laminar airflow cabinets, and a pharmaceutical organic lab. VOC reduction was measured by percentage removal, leaf area, and total weight absorbed. The initial concentration for each VOC was ranged between 10 and 8 ppm for (benzene, toluene, benzaldehyde, and acetophenone). The leaf area per plant species was determined using ImageJ software and ranged between 771.39 and 1471.77 cm^2^. The exposure duration was standardized at 40 min, corresponding to a realistic laboratory session *Cordyline fruticosa* showed the highest VOC removal efficiency at 87.5%, reducing the mean VOC concentration from 2.92 ± 0.54 ppm to 0.36 ± 0.14 ppm. *Syngonium podophyllum* followed with an 81.69% removal efficiency, reducing VOCs to 0.72 ± 0.17 ppm. *Epipremnum aureum and Chlorophytum comosum* showed removal efficiencies of 77.23% and 62.5%, respectively. *Cordyline fruticosa and Syngonium podophyllum* can reduce TVOC, CO_2_, CO, PM_2.5_, and PM_10_ levels by 87.5%, 88.23%, 36.78%, 100%, and 100%, respectively *.*A combined application of *Cordyline fruticosa* and *Syngonium podophyllum* in Plant-Based Bio-Filters (PBBFs) significantly improved indoor air quality. These findings highlight the potential of AP as a natural, cost-effective solution for VOC mitigation, offering a practical approach to environmental management and sustainable indoor design in laboratory settings.

**Supplementary Information:**

The online version contains supplementary material available at 10.1038/s41598-025-30145-8.

## Introduction

Urban civilization has a great effect on the surroundings and human health. The level of indoor air pollutants may be exceed from two to five times than the outdoors air pollutants^1^. One of the indoor environments is a pharmaceutical laboratory as a source of indoor pollution but the exact contribution of elimination of indoor air pollution remains incompletely understood and become a major concern, that students at the pharmaceutical laboratories as organic chemistry lab and pharmaceutics lab are dealing with some volatile organic compounds (VOCs) during their studying five years exposed to these compounds and particulate matter. The most common VOC’s which emits from different laboratory experiments and methods are vary in levels of pollution, Some of the volatile organic compounds used or produced in these experiments may be released into the atmosphere, which causes air pollution, such as (benzene, toluene, ethylbenzene and xylene) BTEX, alcohols, ketones, aromatic hydrocarbons, halo-hydrocarbons, amines, esters, ether, aldehyde as formaldehyde, chloroform, trichloroethylene^2^. Therefore, the scope of investigation of elimination of indoor air pollutants in pharmaceutical laboratories is increasingly concerned.

The health effects from VOC’s exposure can result from exposure to low concentrations of VOCs over both short and long temporal periods. Life time exposure to certain VOCs, such as benzene and 1,3- butadiene, at concentrations as low as 1 µg/m^3^, has been associated with increased cancer risk^3,4^. Many strategies and technologies have been developed aiming to reduce or eliminate the emissions of indoor air pollution^5^. Most advanced techniques are chemical based and involve the use of activated carbon for adsorption, ozonization, electrochemical oxidation, nanofiltration, coagulation, chlorination, chemical purification, ventilation, isolation and reverse osmosis^6^.

These technologies, however, are not as efficient as other advanced chemical and physical techniques but can be promising and hold a lot of potential. Especially the emergence of techniques involving plants the process is called “Phytoremediation” removing pollutions by plants. Phytoremediation is an affordable and more ecofriendly, efficient, low-cost, and well-accepted strategy based on plant species to promote environmental restoration means to purify polluted indoor air and their impact on human health. The available literature on phytoremediation, including experimental works for removing (VOCs) and particulate matter from the indoor air and associated challenges and opportunities, are reviewed^1^.

A few plants have the capacity to absorb, corrupt, or adjust harmful poisons through phytovolatilization is frequently considered advantageous. The kind of plant activity being employed in these techniques depends on the nature of the compounds being remediated and the type of plant parts being used in the process^7^. Air pollutant VOCs can be effectively removed by plants. For example, *Brassica* species absorb SO_2_ and NO_2_ from polluted air, *Chenopodium murale* removes volatile hydrocarbons, *Zamioculcas zamiifolia* removes formaldehyde^8^, Trichloroethylene (TCE) and tetrachloroethylene (PCE) removed by *Salix* species^9^. The bio-concentration levels of total VOCs in *Epipremnum aureum* were noted as 74.71 to 174.42, signifying that *E. aureum* is effective for removal of VOCs naturally and sustainably. The tropical *leguminous* tree *Leucaena leucocephala* was studied to treat pharmaceutical contaminants such as ethylene dibromide and trichloroethylene^10^.

Volatile Organic Compounds (VOCs) Chlorinated solvents such as tetrachloroethylene and trichloroethylene were effectively degraded by tree cores. Were used as test compounds^7^. Direct phytovolatilization involves a simple pathway in which pharmaceutical chemicals are taken up by the plant and translocate, to be volatized from the stem, trunk, and leaves by transpiration. These substances may diffuse through the epidermis and cutin layers because they are mildly hydrophobic. Plants remove airborne pollutants through multiple physiological and physical mechanisms, which together contribute to their effectiveness as natural biofilters. The primary pathways include stomatal uptake and diffusion of gaseous compounds through leaf pores, adsorption onto leaf surfaces and cuticular wax layers. Additionally, plants capture particulate matter (PM) through surface roughness, trichomes, and epicuticular wax, which enhance dust adhesion and retention. These mechanisms vary among species, depending on leaf morphology, stomatal density. Recent studies have provided in-depth mechanistic insights into these processes, highlighting the importance of both the phyllosphere and rhizosphere in VOCs removal and PM capture Indirect phytovolatilization can increase the flow of these volatile pollutants^9^.

There are challenges in green buildings, such as creating an effective green environment and providing relaxing surroundings. Air phytoremediation technology has many potential advantages to reduce pollutants in air and improve air quality; they fix carbon dioxide through photosynthesis and help to decrease greenhouse gases in the atmosphere^3^. A list of plants for removing specific pollutants are presented using green wall system or potted plant. It is found that spider plants and *philodendron* show high potential to remove formaldehyde, while *Chlorophytum comosum* can remove PMs. Among the newest types of phytoremediation systems that incorporate the home’s heating, cooling, and ventilation are active living walls. Furthermore, there is a need for removing pollution in high concentration by plants, particularly indoor air VOCs pollutants and Particulate matter was not studied in detail especially in pharmaceutical laboratory indoor air and, therefore, more investigations are needed to consider their effect on indoor air quality^1^.

**Fig. 1:**
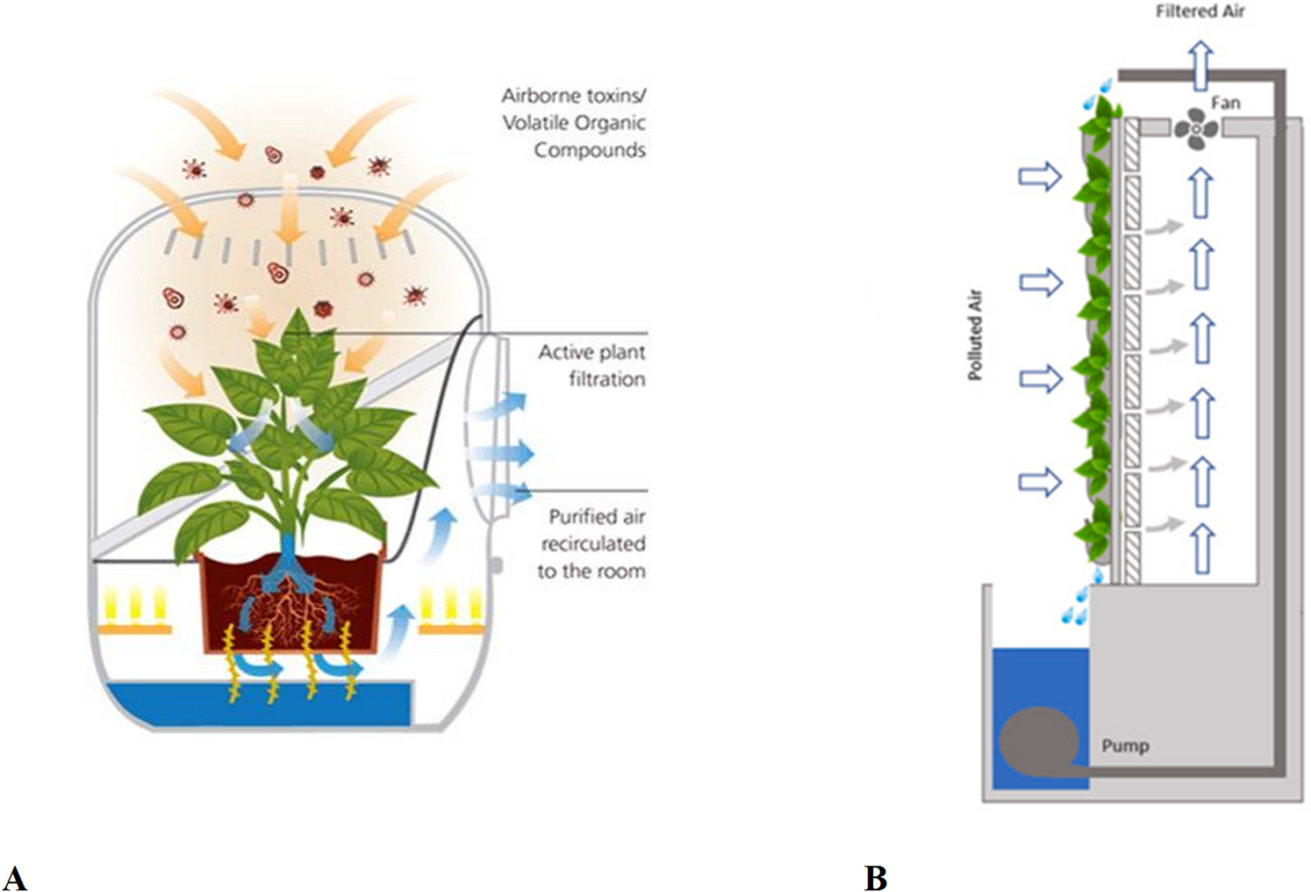
(A) Plant based bio-filter and green wall sourced from https://gajitz.com/clearing-the-air-naturally-plants-technology-scrub-toxins/, and (B)-Green wall (Mannan and Al-Ghamdi^10^).

State-of-the-art ‘Smart Bio-Filter’ illustrated in (Fig. 1), that technology can make breathing fresh, works through the air-purifying natural leafy plant. The laboratory room air interacts with leaves and goes to the soil-root zone where maximum pollutants are purified. This technology is used along with patent-pending “Breathing Roots” to exponentially increase the phytoremediation process of the plants. Effectively improves indoor air quality by removing particulate, gaseous, organic volatile solvents (VOCs) contaminants while increasing the oxygen levels in the indoor space through specific plants.

Reducing indoor pollution in pharmaceutical laboratories can be controlled by combination of biofiltration and phytoremediation results in the bio-wall technique^1^. Further research is needed before this technology becomes widely adopted and implemented within indoor environments. To further validate the potential of these systems for air quality remediation, reproducible laboratory and Feld experimentation is required to quantify the effects and variances with respect to system designs^12^.

The study aimed to investigate indoor Air Quality (IAQ) in pharmaceutical laboratories by using air phytoremediation technology (AP), the study also explored the most efficient plant for indoor air phytoremediation technology (AP) for reducing total volatile organic compounds (TVOCs) and particulate matter (PM) for the sake of proposing an eco-friendly design of plant-based bio-filter using potted plant species and/or green wall.

## Materials and methods

### Experimental chambers and laboratory settings

#### Incubator chamber

**Fig. 2:**
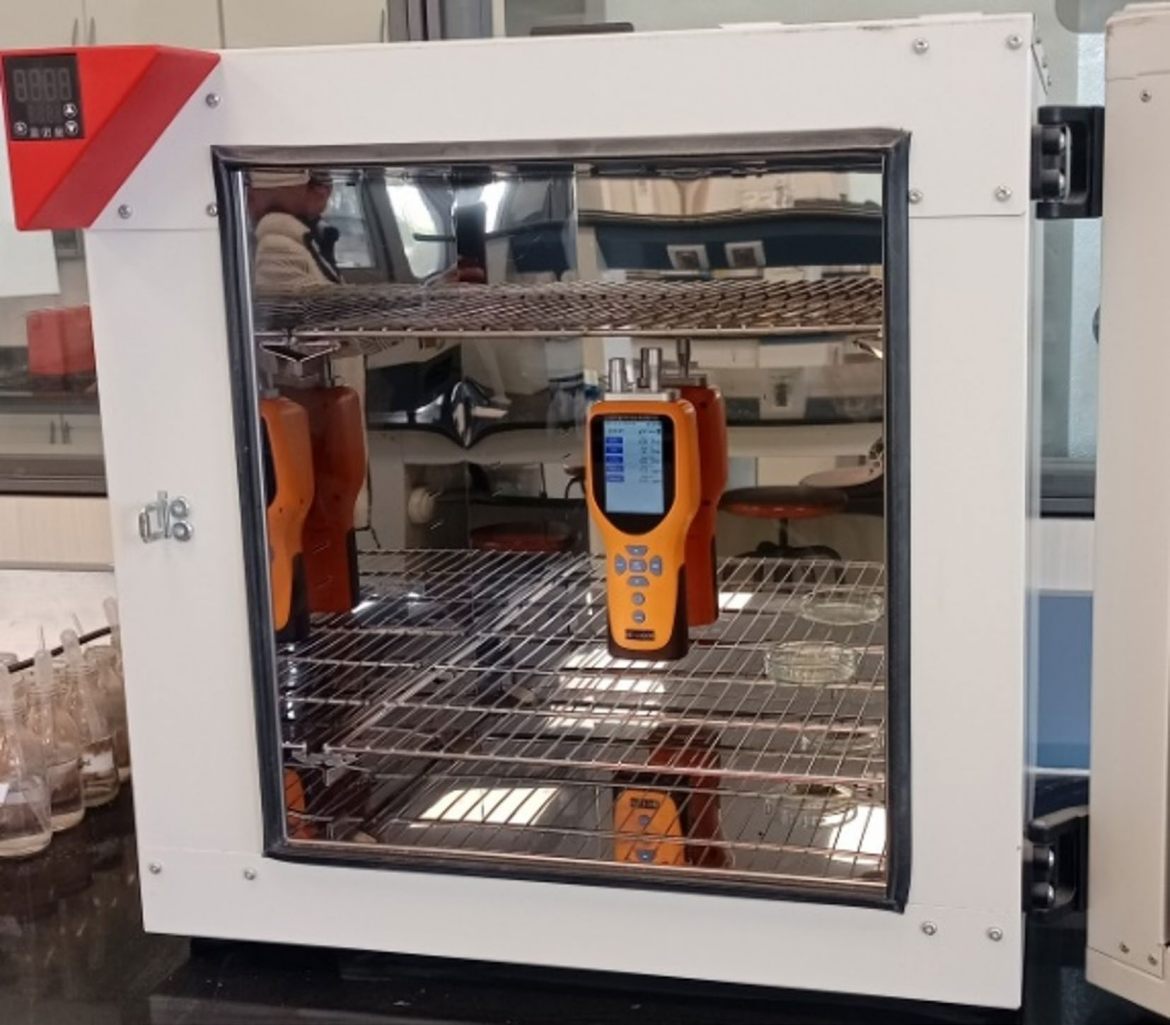
Incubator chamber.

As shown in Fig. 2, the first chamber used to assess the phytoremediation efficiency of the plants was an incubator chamber: (Binder ED-S 056 – Germany) with a capacity of 126 L, measuring 55 cm in length, 51 cm in width, and 45 cm in height.

#### Laminar air flow cabinet

**Fig. 3:**
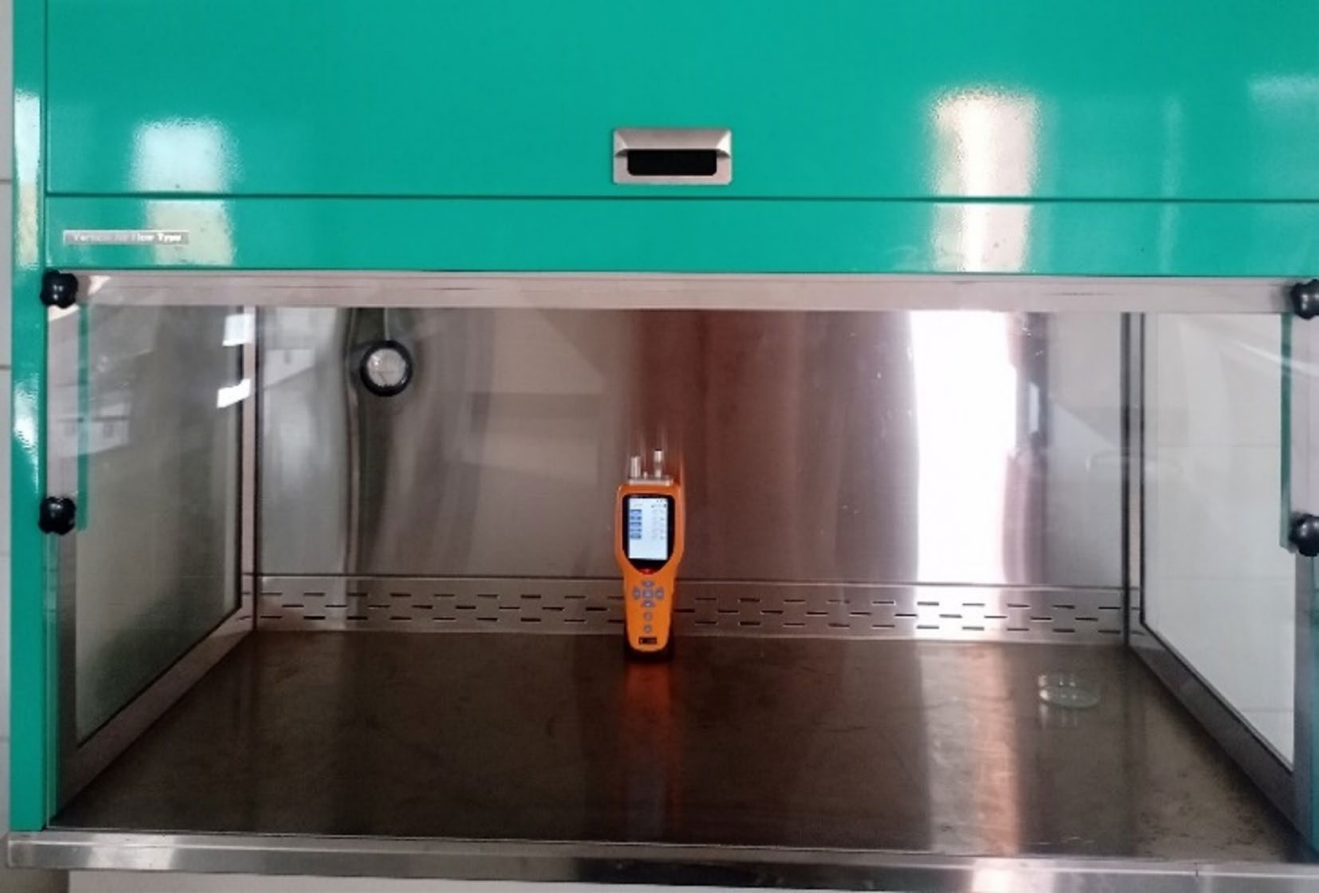
Laminar air flow cabinet.

As shown in Fig. 3, the second chamber was a laminar air flow cabinet: (LabTech LCB-1121VE – Korea) with a volume of 396 L, measuring 120 cm in length, 55 cm in width, and 60 cm in height. This cabinet was equipped with a door on one side, sealed with adhesive rubber insulation tape and secured with adjustable metal clips to prevent air leakage. The system was pre-tested to ensure proper sealing during the experiment.

#### Preparation room

The third set of measurements took place in the preparation room, which had a volume of 59.572 m³, with dimensions of 6.219 m in length, 3.150 m in width, and 3.041 m in height.

#### Pharmaceutical organic laboratory

**Fig. 4:**
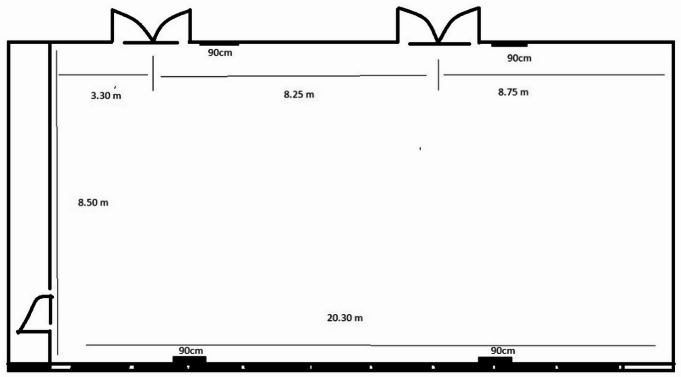
Diagram of organic laboratory.

As shown in Fig. 4, additional real-time measurements were conducted in a real organic chemistry laboratory, with dimensions of 8.5 m in width, 20.34 m in length, and 3.04 m in height, giving a total volume of 525.59 m^3^.

To determine the most effective plant for removing/reducing VOCs from indoor air, using 0.5 mL of benzene, toluene, benzaldehyde, and acetophenone, both with and without plants. Removal efficiency, removal per leaf area, and the weight of VOCs taken were calculated under identical lab conditions. For each experimental setup, a single reading of the VOC concentration was recorded immediately before plant exposure (initial concentration) and again after the 40-minute exposure period (final concentration). Measurements were performed in the incubator chamber, laminar flow cabinet, and preparation room under the same environmental conditions. The detector was calibrated before each test to ensure data accuracy and reliability.

Each plant species was tested in three independent experimental replicates (*n* = 3) for every target VOCs (benzene, toluene, benzaldehyde, and acetophenone). Each replicate consisted of one exposure cycle lasting 40 min under the same environmental conditions. The mean values obtained from the three replicates were used to calculate VOC removal efficiency and for subsequent statistical analyses. For each VOC compound (benzene, toluene, benzaldehyde, and acetophenone), the blank chamber was operated under the same conditions temperature, humidity, light intensity, and exposure duration as the plant-containing chambers. All VOC exposure experiments were conducted under static air conditions to simulate the natural indoor environment and evaluate the passive phytoremediation performance of the plants.

While, to validate the plants’ ability to purify indoor air in real-life conditions, the same plants were used in the Pharmaceutical organic chemistry laboratory experiments, and indoor air quality (IAQ) parameters were measured (VOCs, CO, CO_2_, PM_2.5_ and PM_10_). The removal efficiency (RE%) was also calculated for each experiment.

All experiments were carried out in the mentioned chambers and laboratory settings, both with and without the tested indoor plants. The same experimental conditions used in the previous chambers were consistently applied across all settings.

### VOCs and IAQ measurements

The initial concentrations of volatile organic compounds (VOCs) benzene, toluene, benzaldehyde, and acetophenone were established by fumigating the sealed test chambers with 0.5 mL of each compound using a calibrated micropipette. The injected volume was allowed to evaporate and equilibrate for several minutes to ensure uniform gas-phase distribution before measurement. The resulting vapor concentration was then verified using the Henan OC-1000 Multi-Gas Detector (Oceanus Gas Detector - China, Historical Data V4.2.4)^13^. The VOC concentration (ppm) was recorded each minute until full fumigation (initial concentration Ci) (the maximum ppm detected by the instrument equivalent to 0.5 mL of each VOCs), and after introducing the plants for 40 min (equivalent to the practical time in the lab), the final concentration (C_f_) was measured. For each experimental condition, the initial concentration was recorded immediately before introducing the plant into the chamber or laboratory setup, while the final concentration was measured at the end of the 40-minute exposure period. Each test was conducted under controlled environmental conditions, and the detector was calibrated prior to every experimental session to ensure measurement accuracy.

The removal efficiency and removal per leaf area for the VOCs were calculated in the different chambers and rooms, as well as the number of VOCs absorbed by each plant^14,15^. The temperature and relative humidity of the chambers were recorded using a Henan OC-1000 Multi-Gas Detector, while light intensity, simulating a natural indoor environment, was measured using a Luxmeter mobile application at the start of the experiments^16^. The experiments simulated indoor environments without air conditioning. Prior to VOC exposure tests, all plants were acclimated for four weeks under controlled indoor conditions. The acclimation was conducted in a growth chamber maintained at 25 ± 2 °C, 60 ± 5% relative humidity, and a light intensity of 200 ± 20 µmol m^–2^ s^–1^ using cool-white fluorescent lighting^16^.

#### Removal per leaf area calculations

The concentration of each VOC was recorded over three days, two days in the tested chambers (incubator chamber and laminar flow cabinet) and one day in the laboratory preparation room using one potted plant for each VOC. The initial (Ci) and final (Cf) concentrations of each VOC were measured, and the experiment was repeated for each VOC using four potted plants. Removal per leaf area was calculated using plant leaf area in cm^2^, the total leaf area of each plant was then calculated^17^, and the concentration was expressed as ppm per unit area using equation no. (1)

1 $${\text{Removal per leaf area }}\left( {{\mathrm{ppm}}.{\mathrm{c}}{{\mathrm{m}}^{ - {\mathrm{2}}}}} \right){\text{ }}=\frac{{Ci - Cf}}{A}$$

Where Ci is the initial concentration (ppm), Cf = final concentration (ppm), and A = total leaf area (cm^2^).

#### Weight taken by plants of VOCs

Weight (W) of VOC (benzene, toluene, benzaldehyde and acetophenone) uptake by the examined plants was calculated for the three chambers i.e. incubator chamber, laminar air flow cabinet and the laboratory specialist preparation room by using the Eqs. (2), (3)^14^.

2 $${\mathrm{W}}\,=\,{\text{Dppm }} \times {\text{ }}\left( {\frac{V}{{Mc}}} \right){\text{ }} \times {\text{ }}\left( {\frac{{Mwt}}{{{{10}^6}}}} \right){\mathrm{~}}$$

3 $$\Delta {\mathrm{ppm}}\,=\,\Delta {\mathrm{pp}}{{\mathrm{m}}_{\mathrm{i}}}--{\text{ }}\Delta {\mathrm{pp}}{{\mathrm{m}}_{\mathrm{f}}}$$

Where W is the weight (g) of VOC, V is the volume of the system. Mc is mole concentration of VOC. MW is molecular weight of VOC, Δppm is the difference between the initial concentration and final concentration of each VOC.

#### Removal efficiency

The efficiency of the plants in removing these contaminants was determined by calculating the percentage reduction in concentration. The initial concentration (C_0_) of the contaminants in the lab (without plants) was compared to the final concentration (C_n_) after introducing the plants for the same experiment duration under the same conditions (ventilation, number of students, chemicals used). Therefore, the removal efficiency (RE%) was calculated using the Eq. (4)^14^

4 $${\mathrm{RE}}\% {\text{ }}={\text{ }}\left( {{{\mathrm{C}}_0}\, - \,{\mathrm{Cn}}} \right){\text{ }}/{\text{ }}{{\mathrm{C}}_0} \times {\text{ 1}}00$$

In the Pharmaceutical organic chemistry laboratory measurements of indoor air quality (IAQ) parameters VOCs, CO, CO_2_, PM_2.5_, and PM_10_ were conducted during a practical session focused on aromatic, aldehyde, and ketone identification under ventilation and exhaust air conditions. Twelve plant pots (two per bench) were distributed across six lab benches, and measurements were taken using the Henan OC-1000 Multi-Gas Detector^13^ over a 40-minute period, the 40-minute exposure duration was deliberately chosen to simulate the actual operational and exposure period in the pharmaceutical laboratory during student practical sessions. This timeframe reflects the realistic indoor exposure conditions experienced by students and staff while conducting laboratory experiments.

#### Plant material

**Fig. 5:**
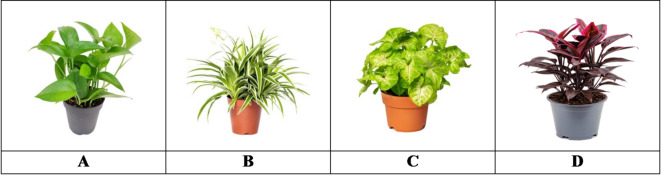
Ornamental indoor studied plants A: *Epipremnum aureum*, B: *Chlorophytum comosum*, C: *Syngonium podophyllum*, D: *Cordyline fruticose*.

Four indoor plant species were utilized in this study presented in Fig. 5. Two of them were recognized as standard air-purifying plants: A: Jade Pothos (*Epipremnum aureum*) from the Araceae family and B: Spider Plant (*Chlorophytum comosum*) from the Asparagaceae family. Additionally, two other plants were tested: C: *Syngonium podophyllum*, also from the Araceae family, and D: *Cordyline fruticosa* from the Asparagaceae family. One pot from each plant species was used in the experiments conducted in the test chambers and the preparation room. A total of twelve pots for each species were sourced from plant suppliers (plant nursery).

The plant species used in this study were obtained from the Arab Academy for Science, Technology & Maritime Transport (AAST) Plant Nursery, Alexandria, Egypt. The plants were taxonomically identified and authenticated by a specialist at the Department of Environmental Studies, Institute of Graduate Studies and Research, Alexandria University. Since the AAST plant nursery does not maintain a voucher specimen collection, no voucher numbers were assigned; however, authenticated live specimens were retained in the nursery for reference during the study period. Collection of the plant materials was conducted with the necessary permissions and in compliance with institutional and national regulations. Appropriate permits were obtained prior to plant collection.

The pots were filled with loamy soil consisting of approximately 30% sand, 30% silt, 15% clay, and 25% humus. This soil was moist, loose, slightly acidic, and rich in microorganisms and nutrients conducive to plant growth. The contribution of soil to VOC absorption was intentionally minimized. Each plant pot was completely sealed at the soil surface using parafilm, leaving only the plant stem exposed. This setup ensured that VOC uptake occurred exclusively through plant tissues (leaves, stems, and roots) via transpiration and metabolic processes, rather than through soil adsorption or microbial degradation. The plants were acclimated to laboratory conditions for one month and watered as needed^18^. Before starting the experiments, the total leaf area and plant height for all species were measured.

The leaves of all plants were classified into three categories: large, medium, and small, and then counted. Three sample leaves from each category group were selected and their surface areas were measured using the ImageJ software (a Java-based processing program developed by the National Institutes of Health and the Laboratory for Optical and Computational Instrumentation)^19^, (version 1.53e, NIH, USA) following a digital image-based method. Three representative leaves from each group were scanned to determine the average surface area (cm²). The total leaf area per plant was calculated by multiplying the mean area of each size group by the number of leaves in that group and summing across all groups.

The total leaf area per plant was calculated using the following Eq. (5):

5 $${{\mathrm{A}}_{{\mathrm{total}}}}=\sum {\left( {{{\mathrm{A}}_{{\mathrm{avg,group}}}} \times {{\mathrm{N}}_{{\mathrm{group}}}}} \right)}$$

Where A_total is the total leaf area per plant, A_avg, group is the average leaf area of each size group (cm^2^), and N_group is the number of leaves in that group. The same potted plants were used throughout all experimental trials; therefore, the number of leaves and total leaf area remained constant during the study period.

### Data analysis

Data analysis was performed using the IBM SPSS software package version 20.0 (Armonk, NY: IBM Corp). Quantitative data were presented as a range (minimum and maximum), along with mean, standard deviation, standard error, median, and interquartile range (IQR). The results were considered statistically significant at a 5% confidence level. The Kruskal-Wallis H test indicated significant differences among the plant groups (*p* < 0.001), demonstrating that incorporating indoor plants impacts the reduction of air pollutants. Data were first tested for normality using the Shapiro–Wilk test (*p* > 0.05). Normally distributed variables were analyzed using one-way ANOVA (F-test), while non-normally distributed variables were evaluated using the Kruskal–Wallis test.

## Results

Table 1 depicted the individual leaf number and leaf area for each pot of studied plant species *Epipremnum aureum*, *Chlorophytum comosum*, *Syngonium podophyllum* and *Cordyline fruticose* where the VOC removal efficiency per leaf area of the four plant species was assessed and their total leaf areas were found to be 771.392, 36.07, 95.038 and 62.779 cm^2^, respectively.

**Table 1:** Plant species leaves number and total leaf area.

	*Epipremnum aureum*	*Chlorophytum comosum*	*Syngonium podophyllum*	*Cordyline fruticosa*
Small leaf no.	11	0	10	3
Small leaf area cm^2^	40.331± 86.01	0	88.773± 85.163	45.571± 86.913
Medium leaf no.	5	12	3	7
Medium leaf area cm^2^	50.989 ± 85.041	32.47± 85.163	93.574± 88.067	56.993 + 85.028
Large leaf no.	1	30	3	5
Large leaf area cm^2^	73.261 ± 85.842	36.071± 89.611	95.038 ± 90.347	62.779± 87.098
Total leaf area cm^2^	771.392 cm^2^	1471.77 cm^2^	1453.566 cm^2^	849.559 cm^2^

Table 2 presented a VOC Removal Efficiency (RE%) of Four Plant Species in the two Test Chambers and Lab. preparation room. One-way ANOVA analysis (*p* < 0.05) revealed significant differences in VOC removal efficiency among the four plant species within each test chamber, as indicated by distinct superscript letters (a, b, c) in Table 2.

**Table 2:** A VOC removal efficiency (RE%) of four plant species in two test chambers and lab. Preparation room.

VOC	Plant species	Total leaf area (cm²)	RE% incubator chamber	RE% Laminar flow chamber	RE% Laboratory preparation room
Benzene	*Epipremnum aureum*	771.39	52.10 ± 2.1b	63.10 ± 1.8b	89.10 ± 3.0a
	*Chlorophytum comosum*	1471.77	58.81 ± 1.6b	64.30 ± 2.0b	59.42 ± 1.9c
	*Syngonium podophyllum*	1453.57	52.70 ± 1.4b	53.73 ± 2.3c	44.39 ± 1.6c
	*Cordyline fruticosa*	849.56	72.00 ± 2.5a	84.50 ± 2.8a	54.90 ± 1.8b
Toluene	*Epipremnum aureum*	771.39	43.90 ± 1.8a	65.80 ± 2.1a	60.89 ± 1.9b
	*Chlorophytum comosum*	1471.77	41.50 ± 2.0a	47.90 ± 1.6c	87.61 ± 2.5a
	*Syngonium podophyllum*	1453.57	48.00 ± 1.5a	46.00 ± 1.7c	88.96 ± 2.6a
	*Cordyline fruticosa*	849.56	63.55 ± 2.4b	47.50 ± 1.8bc	68.42 ± 2.2b
Benzaldehyde	*Epipremnum aureum*	771.39	42.14 ± 1.3b	74.55 ± 2.2a	45.45 ± 1.7b
	*Chlorophytum comosum*	1471.77	20.75 ± 1.2c	46.70 ± 1.9c	60.60 ± 2.3a
	*Syngonium podophyllum*	1453.57	57.60 ± 1.8a	44.97 ± 1.5c	66.29 ± 2.1a
	*Cordyline fruticosa*	849.56	23.60 ± 1.4c	74.47 ± 2.3a	74.24 ± 2.5a
Acetophenone	*Epipremnum aureum*	771.39	45.00 ± 1.7b	66.17 ± 1.9b	68.01 ± 2.1b
	*Chlorophytum comosum*	1471.77	53.10 ± 1.8b	54.22 ± 1.8c	74.86 ± 2.4a
	*Syngonium podophyllum*	1453.57	58.90 ± 2.1b	78.96 ± 2.5a	74.81 ± 2.2a
	*Cordyline fruticosa*	849.56	59.00 ± 2.0a	80.28 ± 2.6a	86.50 ± 2.8a

Where: Values are expressed as mean ± SD (*n* = 3). Different superscript letters within each VOC and chamber indicate significant differences among species (one-way ANOVA, *p* < 0.05). Bold values represent the highest removal efficiency observed for each VOC in a given chamber.

### VOCs concentration measurements

#### VOCs conc. measurements taken in incubator chamber

**Fig. 6:**
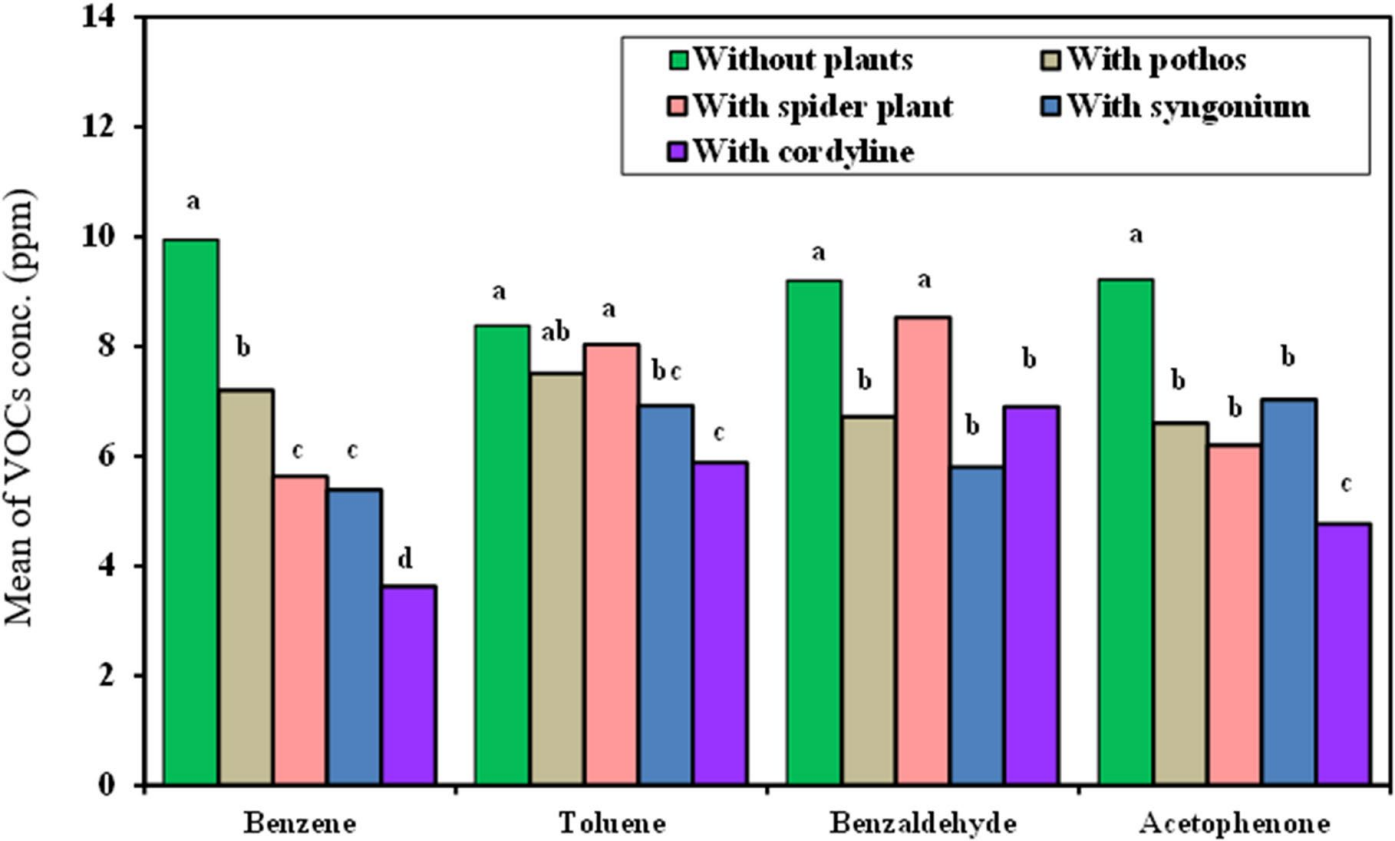
Comparison between the VOCs concentration with and without examined plant in Incubator chamber.

The VOCs RE% of the four examined plants exposed in the incubation chamber demonstrated that the removal of Benzene was best achieved by *Cordyline fruticosa* having RE% of 72%) followed by *Epipremnum aureum*, *Syngonium podophyllum and Chlorophytum comosum possessing* RE% of 52.1, 52.7, and 58.81%, respectively as showed in Fig. 6.

1. Removal of Toluene: *Cordyline fruticosa* exhibited also the greatest RE% (3.55%), followed by *Epipremnum aureum*, *Syngonium podophyllum Chlorophytum comosum* which showed RE% of 43.9, 48, and 41.5%, respectively.
2. Removal of Benzaldehyde: *Syngonium podophyllum* has the greatest RE% (57.6%), followed by *Epipremnum aureum*, *Cordyline fruticosa* and *Chlorophytum comosum* which showed RE% of 42.14%, 23.6% and 20.75% respectively.
3. Removal of Acetophenone: *Cordyline fruticosa* exhibits the greatest RE% (59%) and is closely followed by *Epipremnum aureum*, *Syngonium podophyllum*, and *Chlorophytum comosum* which showed RE% 58.9%, 53.1% and 45% respectively.

The statistical analysis shows that, in comparison to the control (no plants), all four plants considerably decreased the concentrations of the VOCs under study in the incubator chamber. For every VOC examined, *Cordyline fruticosa* consistently demonstrated the highest removal efficiency, with *Syngonium podophyllum*, *Chlorophytum comosum*, and *Epipremnum aureum* following closely behind.

#### VOCs conc. measurements in laminar air flow cabinet

**Fig. 7:**
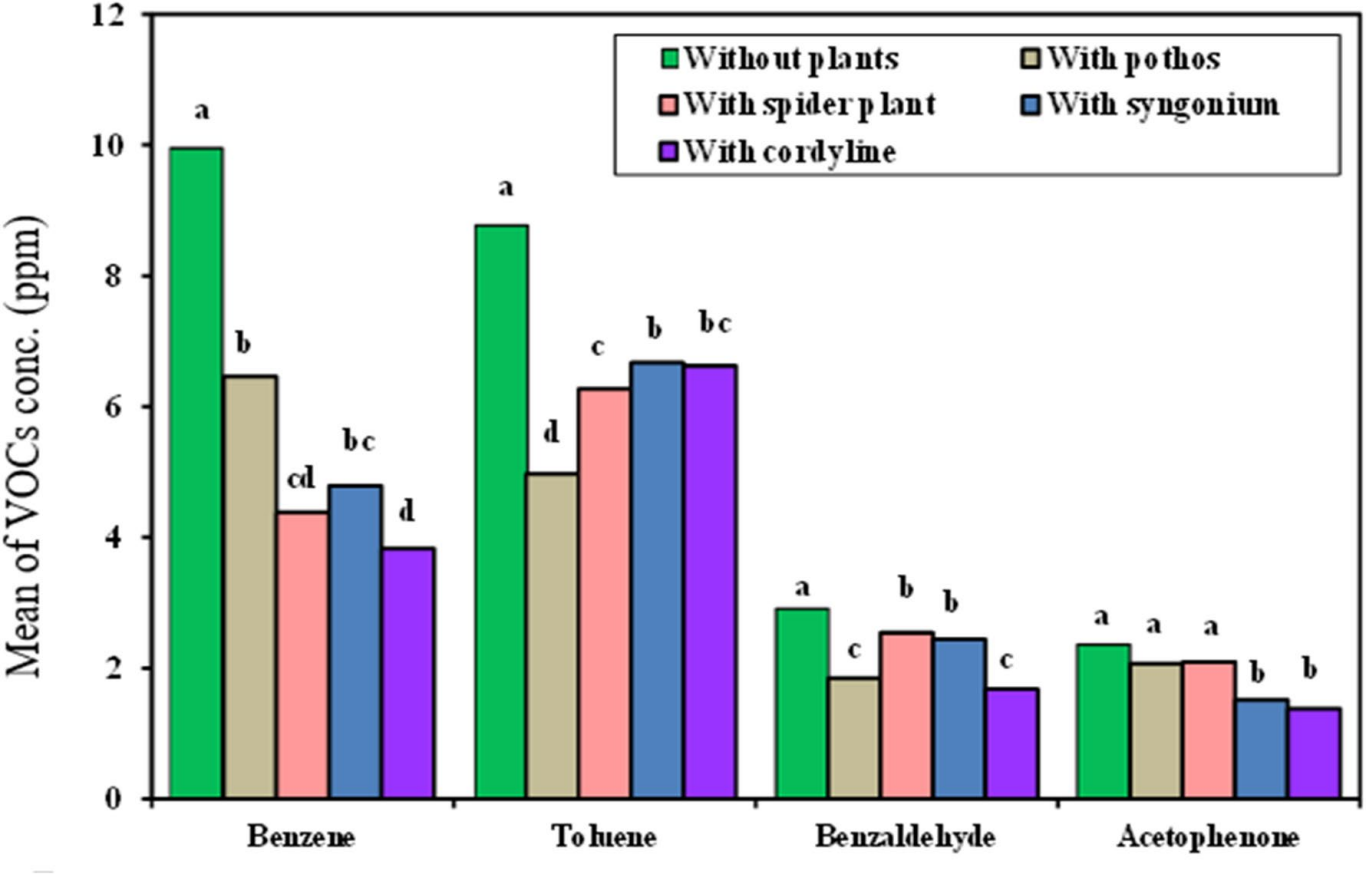
Comparison between the VOCs concentration with and without examined plant in laminar air flow cabinet.

As demonstrated in (Fig. 7), the VOCs RE% data for the four examined plants exposed in laminar air flow cabinet pointed out that *Cordyline fruticosa* exhibited the highest RE% of benzene at 84.5%,

1. Benzene Removal: followed by *Chlorophytum comosum* of 64.3%, *Epipremnum aureum* of 63.1%, and *Syngonium podophyllum* at 53.73%.
2. Toluene Removal: *Epipremnum aureum* shows the highest RE% for toluene of 65.8%, followed by *Chlorophytum comosum* of 47.9%, *Cordyline fruticosa* of 47.5%, and *Syngonium podophyllum* at 46%.
3. Benzaldehyde Removal: *Epipremnum aureum* and *Cordyline fruticosa* show high RE%s for benzaldehyde (74.55% and 74.47%, respectively), with *Chlorophytum comosum* and *Syngonium podophyllum* following closely behind at 46.7% and 44.97%, respectively.
4. Acetophenone Removal: *Cordyline fruticosa* shows the highest RE% for acetophenone at 80.28%, followed closely by *Syngonium podophyllum* at 78.96%, *Epipremnum aureum* at 66.17%, and *Chlorophytum comosum* at 54.22%.

Conclusively, *Cordyline fruticosa* demonstrated the highest removal effectiveness for all VOCs examined in both chambers. When the plant’s VOC concentrations were compared to those of the other plants and the control (which had no plants), they were much lower. In the incubation chamber, *Cordyline fruticosa* > *Syngonium podophyllum* > *Chlorophytum comosum* > *Epipremnum aureum* was the order of efficiency for the elimination of benzene and toluene. Although the exact ranks varied slightly, *Cordyline fruticosa* was still the most effective in the laminar flow chamber overall.

The best VOCs elimination effectiveness was demonstrated by *cordyline fr*uticosa, especially for acetophenone and benzene. *Syngonium* and *Chlorophytum comosum* also demonstrated significant reductions but to a lesser degree than *Cordyline fruticosa*. *Epipremnum aureum* nevertheless helped reduce volatile organic compounds. These results confirm that participating in certain plant species, particularly *Cordyline fruticosa*, into interior space environments can substantially improve air quality by reducing VOC concentrations.

#### Laboratory specialist preparation room VOCs measurements

**Fig. 8:**
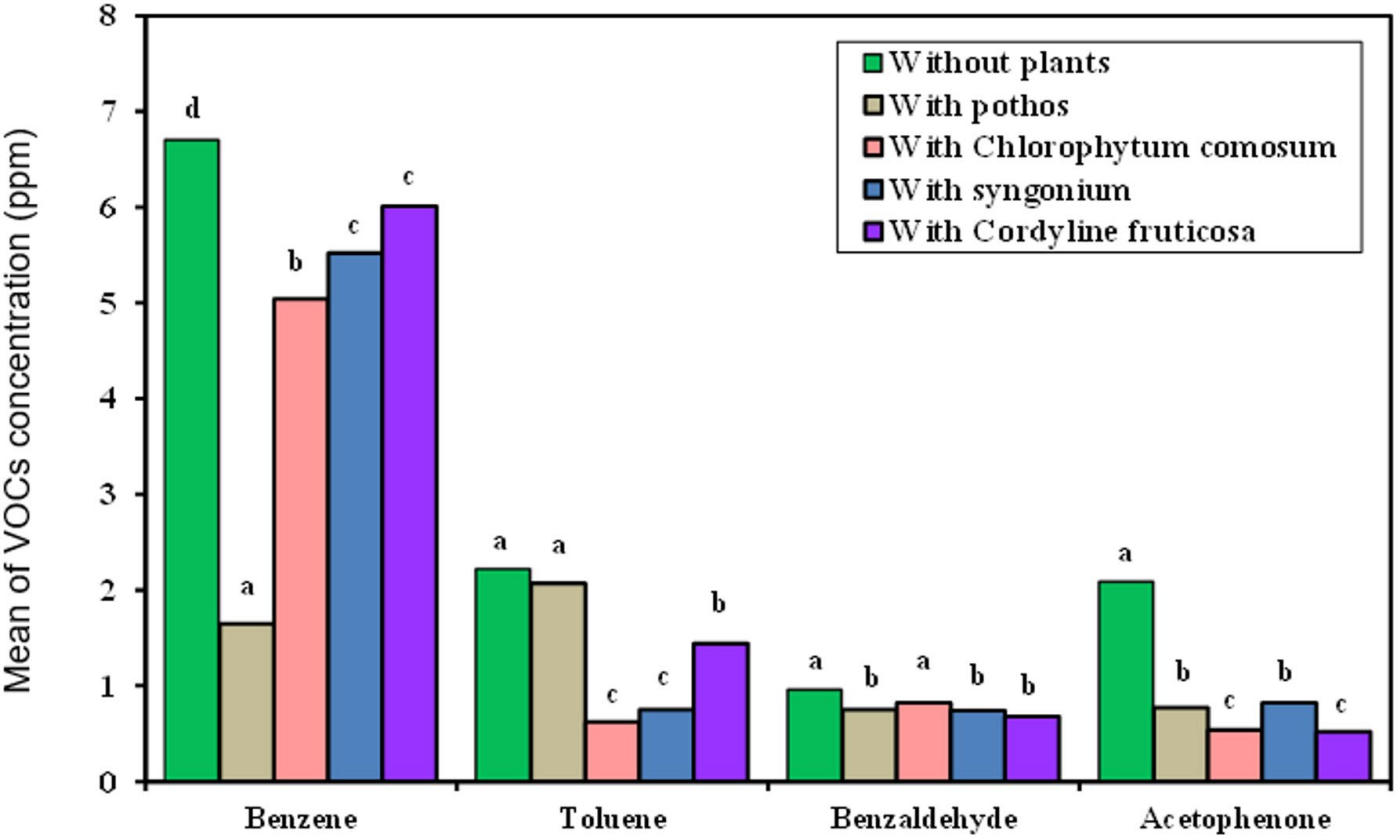
Comparison between the VOCs concentration with and without examined plant in laboratory preparation room.

As demonstrated in (Fig. 8) the data provided represents evolution of the VOCs RE% for the four examined plants exposed in incubator chamber. The following are a summary of important observations:

1. Benzene Removal: *Epipremnum aureum* has the highest RE% of 89.1%, followed by *Chlorophytum comosum* of 59.42%, *Cordyline fruticosa* of 54.9%, and *Syngonium podophyllum* of 44.39%.
2. Toluene Removal: *Syngonium podophyllum* shows the highest RE% for toluene at 88.96%, followed closely by *Chlorophytum comosum* at 87.61%, *Cordyline fruticosa* at 68.42%, and *Epipremnum aureum* at 60.89%.
3. Benzaldehyde Removal: *Cordyline fruticosa* has the highest RE% for benzaldehyde at 74.24%, followed by *Syngonium podophyllum* at 66.29%, *Chlorophytum comosum* at 60.60%, and *Epipremnum aureum* at 45.45%.
4. Acetophenone Removal: *Cordyline fruticosa* shows the highest RE% for acetophenone at 86.50%, followed by *Chlorophytum comosum* at 74.86%, *Syngonium podophyllum* at 74.81%, and *Epipremnum aureum* at 68.01%.

Further *evidence from this investigation demonstrates the great efficacy of Cordyline fruticosa and Syngonium* podophyllum in eliminating volatile organic compounds (VOCs) from the interior air of laboratory specialty preparation rooms, which makes them useful for enhancing indoor air quality in a variety of settings.

#### Removal per leaf area

Similarly, statistical analysis using one-way ANOVA (*p* < 0.05) confirmed significant differences among plant species for both removal per leaf area and total weight of VOCs absorbed in each chamber, as denoted by superscript letters (a, b, c) in Table 3. These findings highlight distinct phytoremediation capacities of the tested species under varying environmental conditions.

**Table 3:** VOCs removal per leaf area and weight of VOCs taken by four plant species in two test chambers and lab. preparation room.

VOC	Plant species	Removal per leaf area Incubator (×10^–3^) (ppm·cm^–2^)	Removal per leaf area Laminar flow (×10^–3^) (ppm·cm^–2^)	Removal per leaf area Lab. prep room (×10^–3^) (ppm·cm^–2^)	Weight of VOCs taken incubator (g)	Weight of VOCs taken laminar flow (g)	Weight of VOCs taken Lab. prep room (g)	Total leaf area (cm^2^)	Significance (ANOVA, *p*<0.05)
Benzene	*Epipremnum aureum*	2.9c	8.82b	11.00a	0.0039c	0.0376b	7.005a	771.39	a, b,c
	*Chlorophytum comosum*	3.06c	4.37c	2.15ᵈ	0.0079b	0.0249c	2.640b	1471.77	a, b,c
	*Syngonium podophyllum*	4.66b	3.25c	2.96c	0.0119b	0.0373b	3.590b	1453.57	a, b,c
	*Cordyline fruticosa*	8.47a	9.95a	6.46b	0.0126b	0.0397b	4.573b	849.56	a, b,c
Toluene	*Epipremnum aureum*	7.70b	8.52b	1.62c	0.0147b	0.0510b	1.476c	771.39	a, b,c
	*Chlorophytum comosum*	3.17c	3.25c	2.64c	0.0115b	0.0372b	4.556b	1471.77	a, b,c
	*Syngonium podophyllum*	3.30c	3.16c	2.83c	0.0118b	0.0357b	4.814b	1453.57	a, b,c
	*Cordyline fruticosa*	5.98b	5.59b	3.61b	0.0125b	0.0368b	3.596b	849.56	a, b,c
Benzaldehyde	*Epipremnum aureum*	7.32b	3.26c	7.70b	0.0154b	0.0217b	0.776c	771.39	a, b,c
	*Chlorophytum comosum*	3.70c	1.15ᵈ	8.21b	0.0149b	0.0145c	1.565b	1471.77	a, b,c
	*Syngonium podophyllum*	3.10c	1.33c	1.03c	0.0123b	0.0165b	1.940b	1453.57	a, b,c
	*Cordyline fruticosa*	3.92c	2.92b	1.73c	0.0091c	0.0212b	1.901b	849.56	a, b,c
Acetophenone	*Epipremnum aureum*	7.32b	3.30c	1.96c	0.0200b	0.0284b	2.542b	771.39	a, b,c
	*Chlorophytum comosum*	2.51c	1.35ᵈ	9.76b	0.0131b	0.0221b	3.333b	1471.77	a, b,c
	*Syngonium podophyllum*	3.25c	1.91c	1.46c	0.0168b	0.0309b	3.585b	1453.57	a, b,c
	*Cordyline fruticosa*	6.25b	3.44b	2.98b	0.0188b	0.0326b	4.259b	849.56	a, b,c

Where: Values are expressed as means of three replicates (*n* = 3). Different superscript letters (a, b, c, d) within each VOC and chamber indicate significant differences among species (one-way ANOVA, *p* < 0.05). ‘Removal per leaf area’ reflects VOC absorption normalized by total leaf area, while ‘Weight of VOCs taken’ represents the total VOC mass absorbed per exposure. Bold values (in original figure) highlight the highest efficiency values for each VOC and chamber.

#### Removal per leaf area in incubator chamber

As demonstrated in Table 3*Cordyline fruticosa* demonstrated the highest benzene removal per leaf area, while *Epipremnum aureum* excelled in removing toluene, benzaldehyde, and acetophenone.

#### Removal per leaf area in laminar flow chamber

***Cordyline fruticosa*** showed the highest removal rate per leaf area for benzene and acetophenone, while ***Epipremnum aureum*** was most effective for toluene and benzaldehyde removal.

#### Removal per leaf area in laboratory specialist preparation room

Our study demonstrated that ***Cordyline fruticosa*** was most successful in removing toluene, whereas ***Epipremnum aureum*** demonstrated the highest removal rate per leaf area for benzene. ***Chlorophytum comosum*** was quite effective at getting rid of acetophenone and benzaldehyde.

#### Weight of VOC taken by examined plants

As demonstrated in Table 2 The analyzed data provided the efficiency of removal of benzene, toluene, benzaldehyde, and acetophenone among the four indoor plant species (*Epipremnum aureum*, *Chlorophytum comosum*, *Syngonium podophyllum*, and *Cordyline fruticosa*) across different study volume chambers and laboratory preparation room for the removal percentages and removal rate per leaf area and weight of VOCs taken by the examined plants.

***Cordyline fruticosa*** consistently showed the best removal efficiency across numerous VOCs in the incubator and laminar flow chambers, especially for benzene and acetophenone, while not as successful as *Cordyline fruticosa*, *Syngonium* and *Chlorophytum comosum* (spider plant) also showed noteworthy removal efficiency. The effectiveness of *Epipremnum aureum* (Pothos) varied; in the laboratory preparation room, it was quite efficient in eliminating benzene, but less so in other situations, such toluene, where it raised rather than decreased the concentration. In the laboratory preparation room, *Epipremnum aureum* and *Syngonium podophyllum* performed better in reducing benzene and toluene. When it came to decreasing acetophenone and benzaldehyde, *Cordyline fruticosa* performed better than *Chlorophytum comosum*. Overall, these findings highlight that these plant species are highly effective in improving indoor air quality by removing harmful VOCs, with each plant having its strengths depending on the specific conditions and VOCs.

### Real laboratory measurements for IAQ parameters (VOCs, CO, CO_2_, PM_2.5_ and PM_10_) without and with examined indoor plants

#### Measurement of organic lab VOCs

**Fig. 9:**
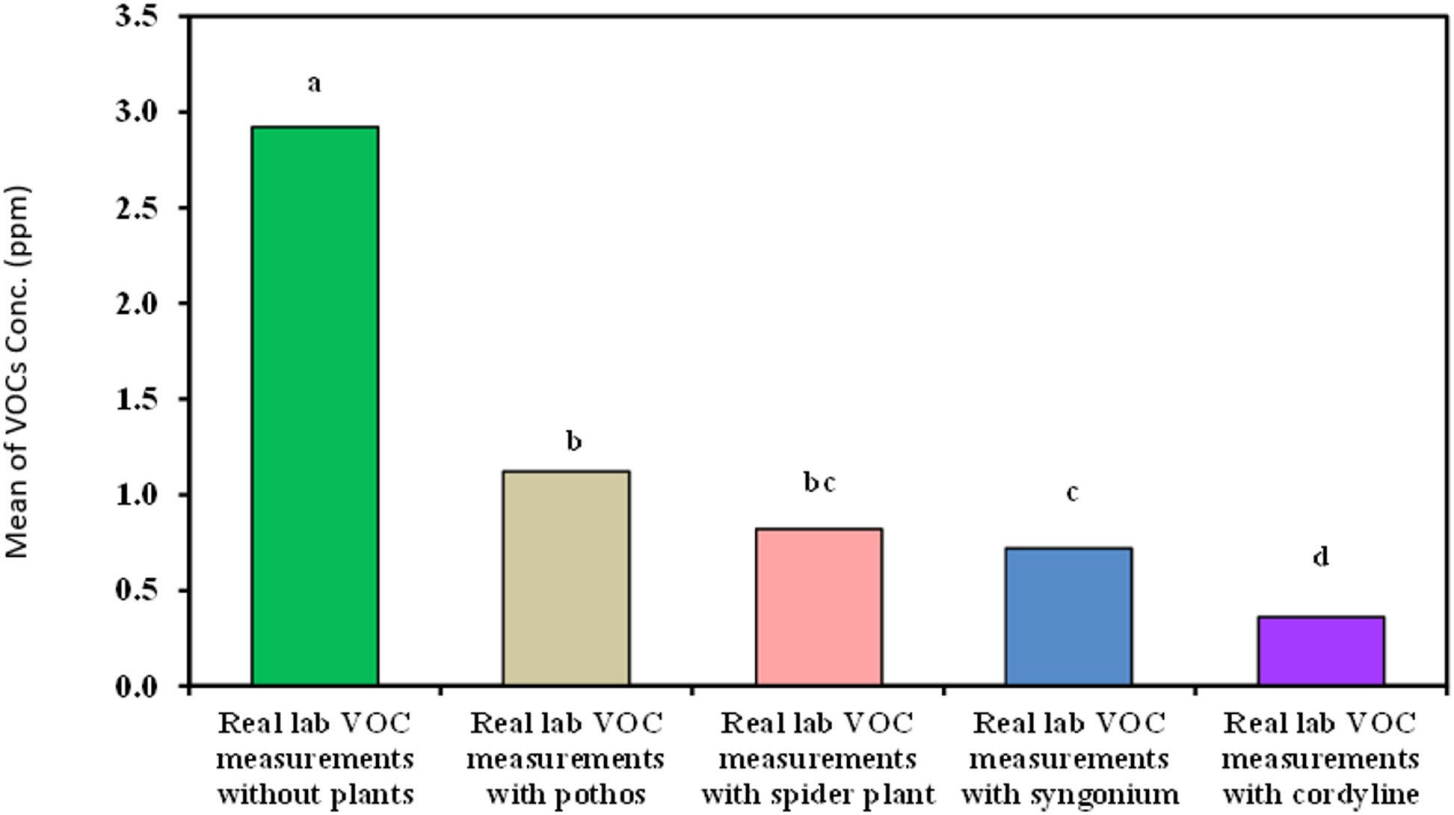
Comparison between real lab VOCs measurements with and without examined plant.

Our study demonstrated in Table 4; Fig. 9 That ***Cordyline fruticosa*** showed the highest VOC removal efficiency at 87.5%, reducing the mean VOC concentration from 2.92 ± 0.54 ppm to 0.36 ± 0.14 ppm. *Syngonium podophyllum* followed with an 81.69% removal efficiency, reducing VOCs to 0.72 ± 0.17 ppm. *Epipremnum aureum* and *Chlorophytum comosum* showed removal efficiencies of 77.23% and 62.5%, respectively.

**Table 4:** Comparison between real lab VOCs measurements with and without examined plant.

Real lab VOC measurements (ppm)	Real lab VOC measurements without plants (*n* = 40)	Real lab VOC measurements with Epipremnum aureum (*n* = 40)	Real lab VOC measurements with Chlorophytum comosum (*n* = 40)	Real lab VOC measurements with Syngonium (*n* = 40)	Real lab VOC measurements with Cordyline fruticosa (*n* = 40)	H	*p*
Min. – Max.	2.10 – 4.31	0.41 – 1.76	0.62 – 1.47	0.51 – 1.0	0.24 – 0.88		
Mean ± SD.	2.92^a^ ± 0.54	1.12^b^ ± 0.44	0.82^bc^ ± 0.19	0.72+ ± 0.17	0.36^d^ ± 0.14	152.82^*^	<0.001^*^
Median	2.92	1.16	0.80	0.63	0.31		
IQR	2.5 – 3.2	0.72 – 1.5	0.65 – 0.94	0.59 – 0.93	0.28 – 0.42		

IQR: Inter quartile range, SD: Standard deviation.

H: H for Kruskal Wallis test, pairwise comparison bet. Each 2 groups were done using Post Hoc Test (Dunn’s for multiple comparisons test).

p: p value for comparing between the three different studied time.

*: Statistically significant at *p* ≤ 0.05.

#### Measurement of organic lab carbon monoxide

**Fig. 10:**
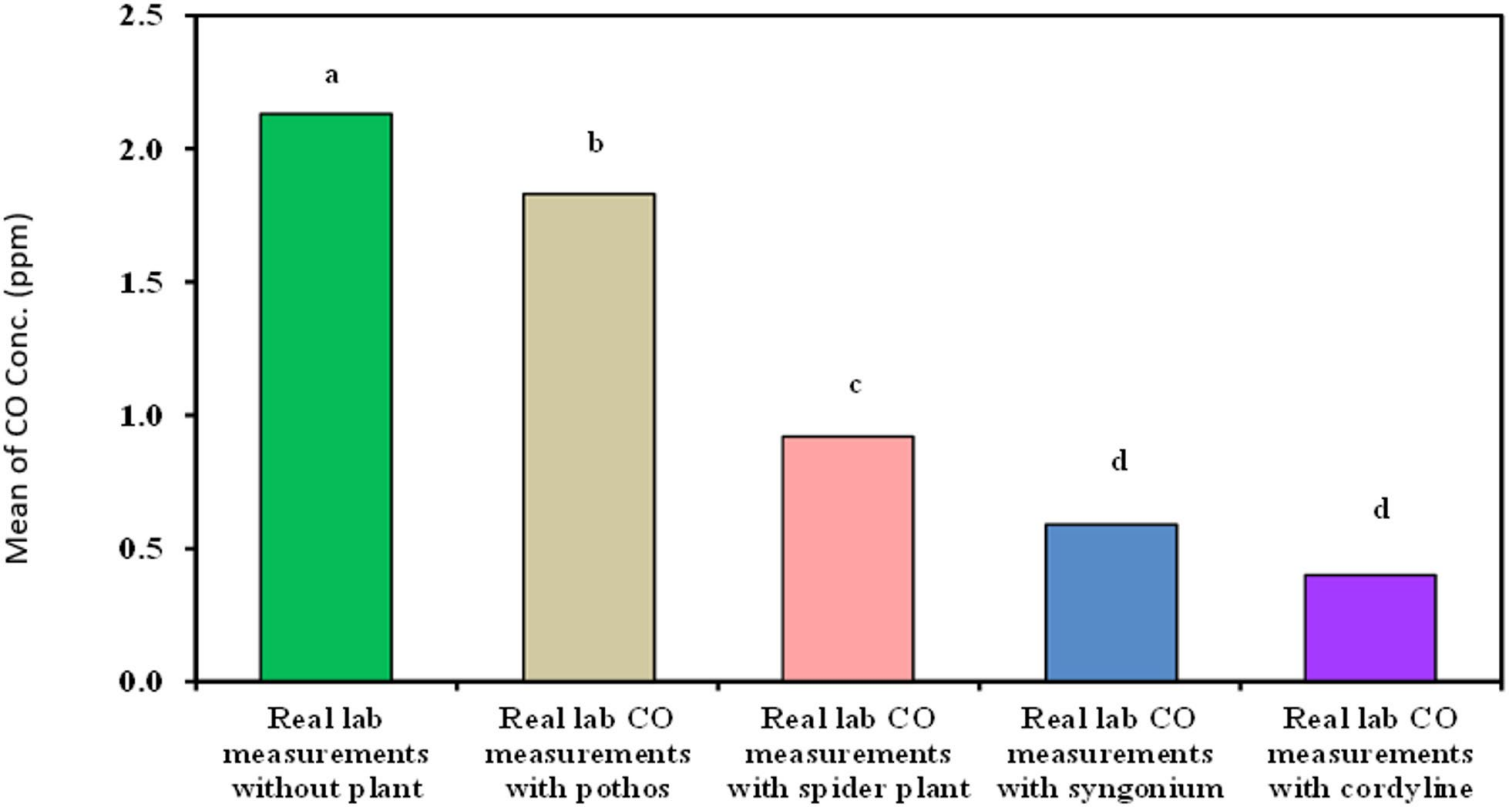
Comparison between real lab CO measurements with and without examined plant.

As demonstrated in Table 5; Fig. 10***Cordyline fruticosa*** showed the best results for decreasing organic lab carbon monoxide with time with removal efficiency 88.23% and mean of 0.40^d^ ± 0.21 followed by *Syngonium*, *Chlorophytum comosum*, *Epipremnum aureum* (70.58%, 11.7% and 11.7% − 0.59^d^ ± 0.13, 0.92^c^ ± 0.20 and 1.83^b^ ± 0.23 respectively).

**Table 5:** Comparison between real lab CO measurements with and without examined plant.

Real lab CO measurements (ppm)	Real lab measurements without plant (*n* = 40)	Real lab CO measurements with Epipremnum aureum (*n* = 40)	Real lab CO measurements with Chlorophytum comosum (*n* = 40)	Real lab CO measurements with Syngonium (*n* = 40)	Real lab CO measurements with Cordyline fruticosa (*n* = 40)	H	*p*
Min. – Max.	1.70 – 2.50	1.30 – 2.40	0.60 – 1.40	0.40 – 0.90	0.10 – 1.10		
Mean ± SD.	2.13^a^ ± 0.22	1.83^b^ ± 0.23	0.92^c^ ± 0.20	0.59^d^ ± 0.13	0.40^d^ ± 0.21	173.43^*^	<0.001^*^
Median	2.10	1.80	0.90	0.55	0.35		
IQR	2.0 – 2.3	1.7 – 2.0	0.70 – 1.0	0.50 – 0.65	0.25 – 0.50		

IQR: Inter quartile range, SD: Standard deviation.

H: H for Kruskal Wallis test, Pairwise comparison bet. Each 2 groups were done using Post Hoc Test (Dunn’s for multiple comparisons test).

p: p value for comparing between the three different studied time.

*: Statistically significant at *p* ≤ 0.05.

#### Measurement of organic lab carbon dioxide

**Fig. 11:**
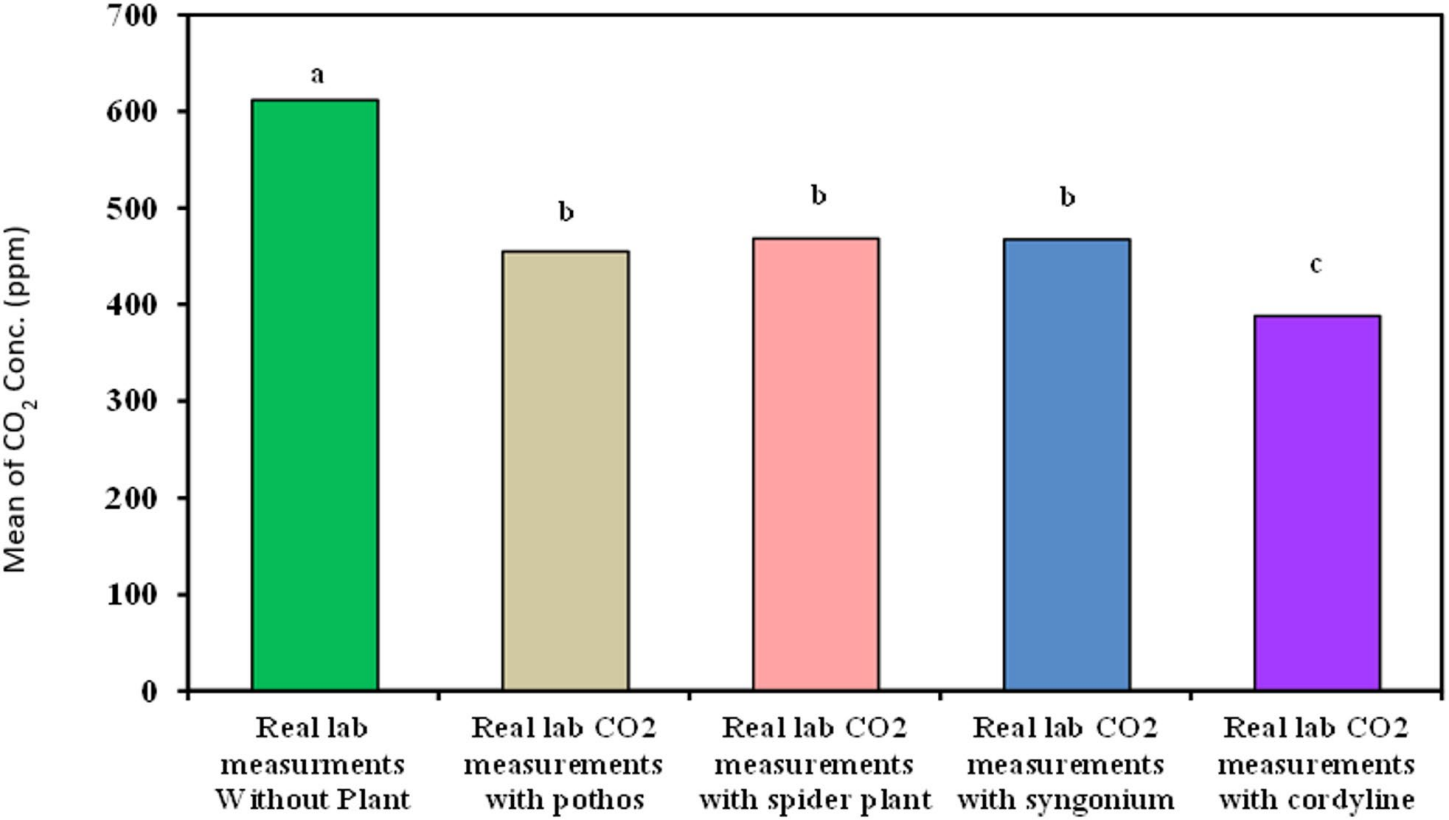
Comparison between real lab CO_2_ measurements with and without examined plant.

As demonstrated in Table 6; Fig. 11***Cordyline fruticosa*** showed the best results for decreasing organic lab carbon dioxide with time with removal efficiency 36.78% and means of 388.2^c^ ± 33.73 followed by, *Syngonium*, *Chlorophytum comosum*, *Epipremnum aureum* (31.27%,25.91% and 20.2% − 467.5^b^ ± 119.0, 468.4^b^ ± 126.2 and 455.2^b^ ± 41.19 respectively).

**Table 6:** Comparison between real lab CO_2_ measurements with and without examined plant.

Real lab CO_2_ measurements (ppm)	Real lab measurements without plant (*n* = 40)	Real lab CO_2_ measurements with Epipremnum aureum (*n* = 40)	Real lab CO_2_ measurements with Chlorophytum comosum (*n* = 40)	Real lab CO_2_ measurements with Syngonium (*n* = 40)	Real lab CO_2_ measurements with Cordyline fruticosa (*n* = 40)	H	*p*
Min. – Max.	535.0 – 700.0	363.0 – 513.0	362.0 – 719.0	352.0 – 688.0	356.0 – 572.0		
Mean ± SD.	611.8^a^ ± 37.87	455.2^b^ ± 41.19	468.4^b^ ± 126.2	467.5^b^ ± 119.0	388.2^c^ ± 33.73	72.415^*^	<0.001^*^
Median	37.87	457.0	412.5	381.0	382.0		
IQR	597.5 – 632.0	443.0 – 488.0	363.0 – 534.5	360.0 – 592.0	375.0 – 394.0		

IQR: Inter quartile range, SD: Standard deviation.

H: H for Kruskal Wallis test, Pairwise comparison bet. Each 2 groups was done using Post Hoc Test (Dunn’s for multiple comparisons test).

p: p value for comparing between the three different studied time.

#### Measurement of organic lab PM_2.5_

**Fig. 12:**
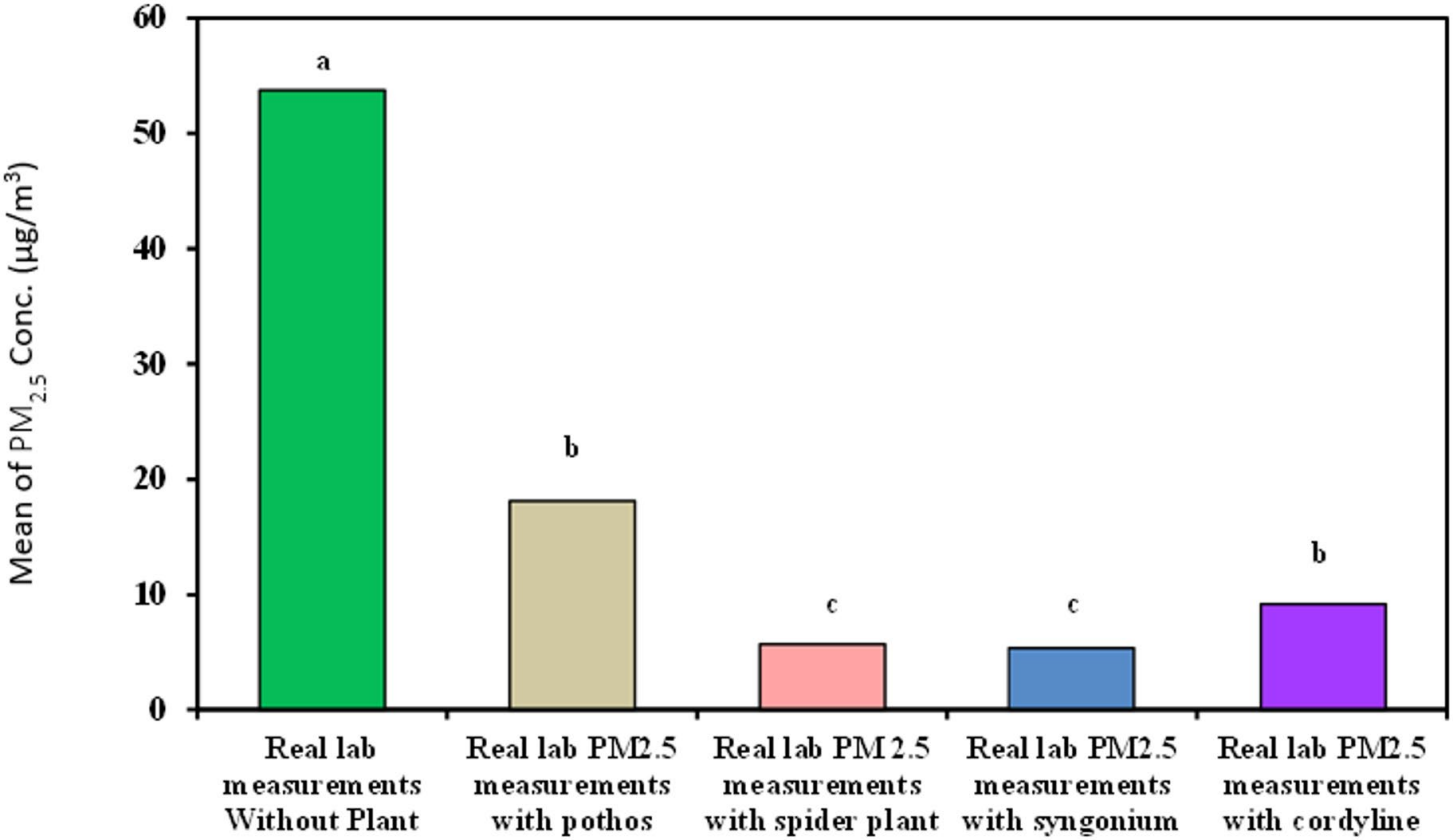
Comparison between real lab PM_2.5_ measurements with and without examined plant.

As demonstrated in Table 7; Fig. 12***Syngonium*** showed the best results for decreasing organic lab PM_2.5_ with time with removal efficiency 100% and means of 5.35^c^ ± 7.49, followed by *Chlorophytum comosum*, *Epipremnum aureum* and *Cordyline fruticosa* (84.61%,64.10% and 53.84% − 5.68^c^ ± 0.83, 9.18^b^ ± 2.43 and 18.12^b^ ± 13.83 respectively).

**Table 7:** Comparison between real lab PM_2.5_ measurements with and without examined plant.

Real lab PM_2.5_ measurements (µg/m^3^)	Real lab measurements without plant (*n* = 40)	Real lab PM_2.5_ measurements with Epipremnum aureum (*n* = 40)	Real lab PM_2.5_ measurements with Chlorophytum comosum (*n* = 40)	Real lab PM_2.5_ measurements with Syngonium (*n* = 40)	Real lab PM_2.5_ measurements with Cordyline fruticosa (*n* = 40)	H	*p*
Min. – Max.	39.0 – 138.0	5.0 – 55.0	3.0 – 7.0	0.0 – 19.0	6.0 – 18.0		
Mean ± SD.	53.73^a^ ± 21.01	18.12^b^ ± 13.83	5.68^c^ ± 0.83	5.35^c^ ± 7.49	9.18^b^ ± 2.43	132.10^*^	<0.001^*^
Median	47.0	15.0	6.0	0.0	8.0		
IQR	42.0 – 56.0	7.5 – 22.5	5.0 – 6.0	0.0 – 14.0	8.0 – 10.0		

IQR: Inter quartile range, SD: Standard deviation.

H: H for Kruskal Wallis test, Pairwise comparison bet. Each 2 groups were done using Post Hoc Test (Dunn’s for multiple comparisons test).

p: p value for comparing between the three different studied time.

*: Statistically significant at *p* ≤ 0.05.

#### Measurement of organic lab PM_10_

**Fig. 13:**
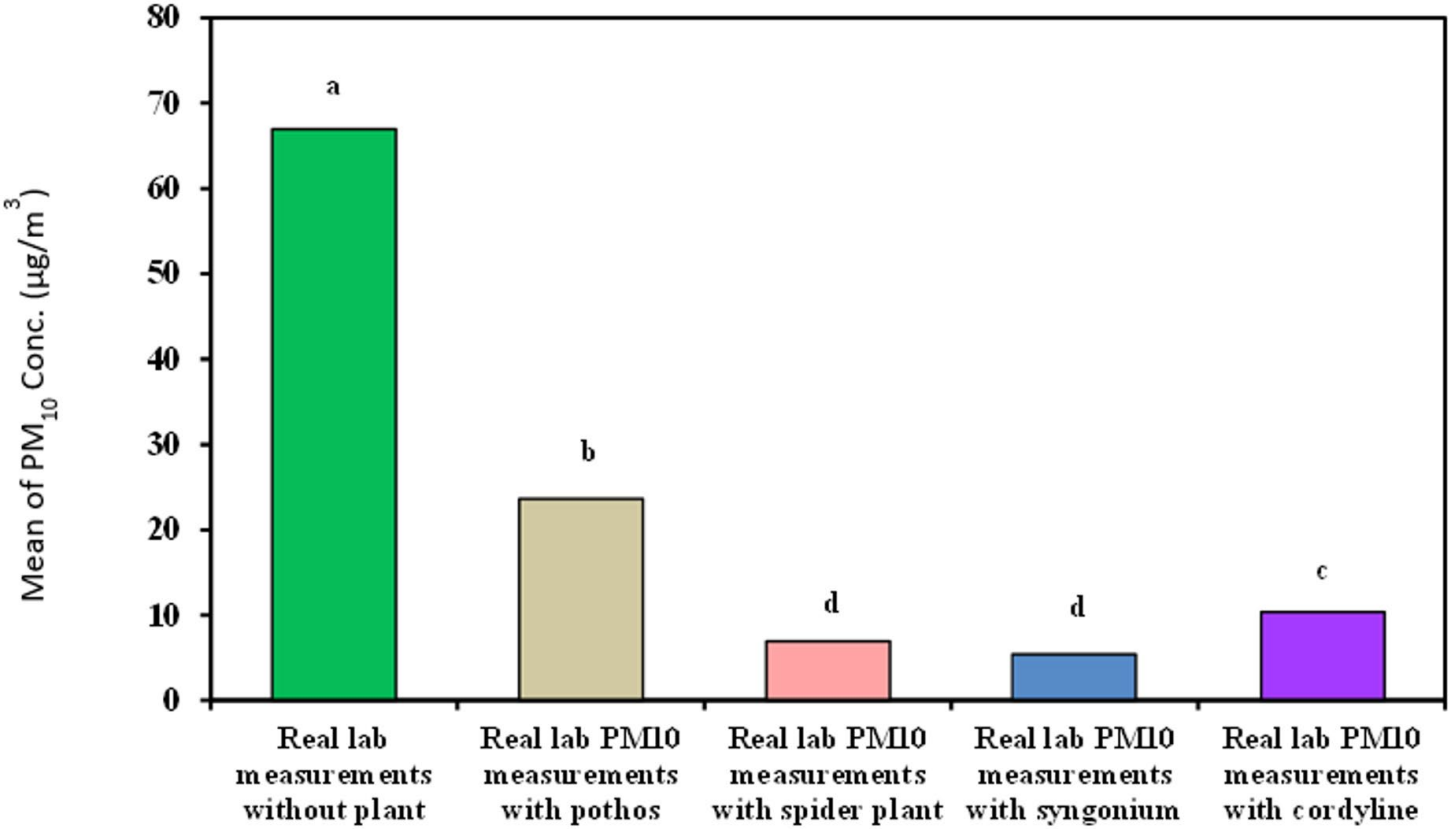
Comparison between real lab PM_10_ measurements with and without examined plant.

As demonstrated in Table 8; Fig. 13***Syngonium*** showed the best results for decreasing organic lab PM_10_ with time with removal efficiency 100% and means of 5.40^d^ ± 6.62 followed by *Chlorophytum comosum*, *Epipremnum aureum* and *Cordyline fruticosa* (88.23%, 66.66% and 60.78% − 6.95^d^ ± 1.58, 10.38^c^ ± 2.87 and 23.65^b^ ± 18.50 respectively).

**Table 8:** Comparison between real lab PM_10_ measurements with and without examined plant.

Real lab PM_10_ measurements (µg/m^3^)	Real lab measurements without plant (*n* = 40)	Real lab PM_10_ measurements with Epipremnum aureum (*n* = 40)	Real lab PM_10_ measurements with Chlorophytum comosum (*n* = 40)	Real lab PM_10_ measurements with Syngonium (*n* = 40)	Real lab PM_10_ measurements with Cordyline fruticosa (*n* = 40)	H	*p*
Min. – Max.	47.0 – 162.0	6.0 – 74.0	4.0 – 11.0	0.0 – 19.0	7.0 – 20.0		
Mean ± SD.	66.95^a^ ± 22.88	23.65^b^ ± 18.50	6.95^d^ ± 1.58	5.40^d^ ± 6.62	10.38^c^ ± 2.87	129.83^*^	<0.001^*^
Median	61.50	18.0	7.0	0.50	9.0		
IQR	54.5 – 70.0	10.5 – 29.0	6.0 – 8.0	0.0 – 12.5	8.0 – 12.5		

IQR: Inter quartile range, SD: Standard deviation.

H: H for Kruskal Wallis test, Pairwise comparison bet. Each 2 groups were done using Post Hoc Test (Dunn’s for multiple comparisons test).

p: p value for comparing between the three different studied time *: Statistically significant at *p* ≤ 0.05.

**Fig. 14:**
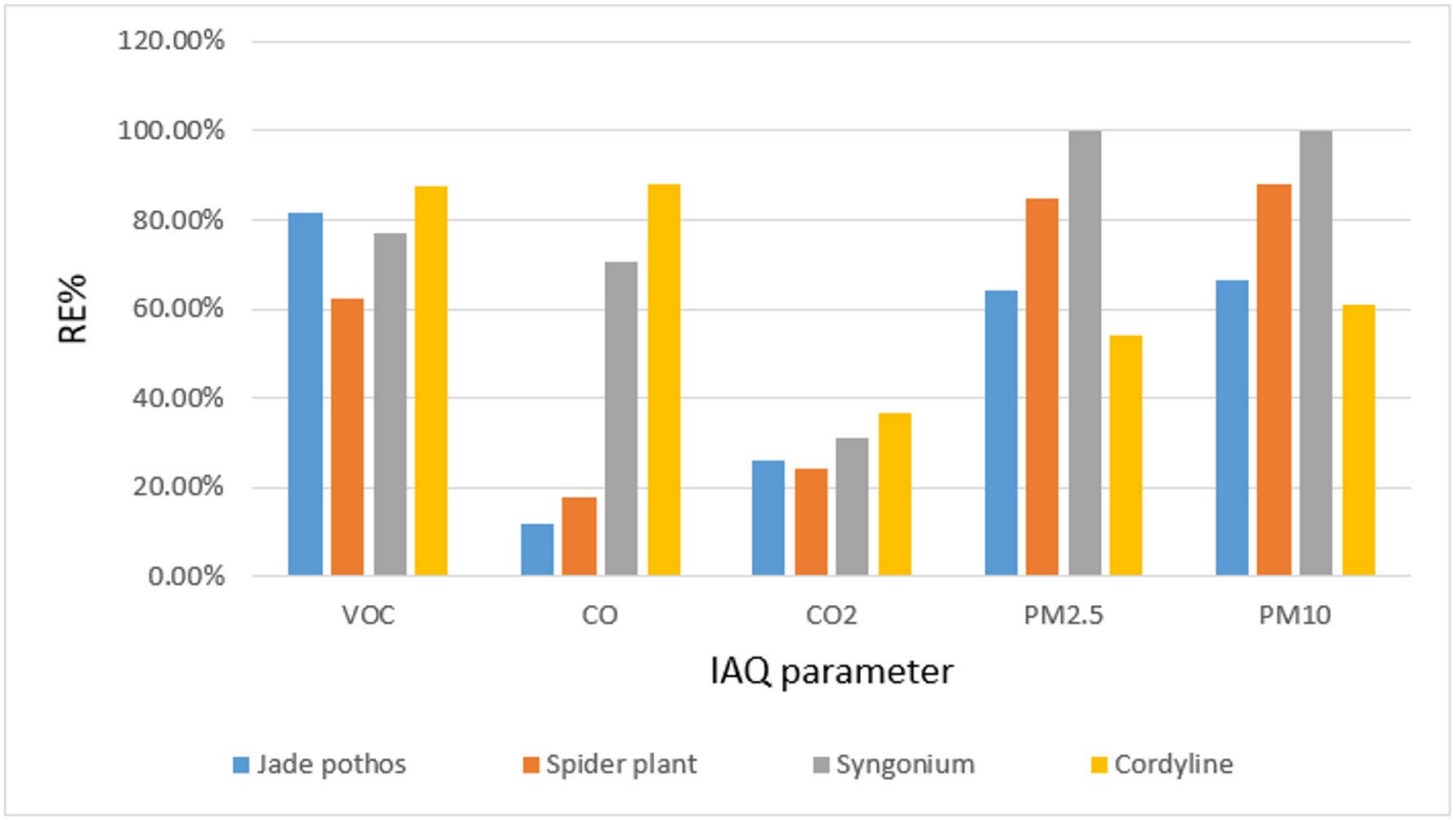
Removal efficiency RE% of examined plant for IAQ parameters in organic Lab.

As demonstrates in Table 9; Fig. 14 Cordyline Fruticosa has the best removal efficiency for VOCs, CO, and CO_2_, demonstrating its potent ability to purify air from these dangerous pollutants and improving overall indoor air quality. Each species of plant has a special advantage when it comes to improving indoor air quality: *Syngonium* and *Chlorophytum comosum* remove particulate matter, and Cordyline fruticose, *Epipremnum aureum* removes CO and VOCs improves IAQ overall.

**Table 9:** Removal efficiency RE% of examined plant for IAQ parameters in organic Lab.

	VOC	CO	CO_2_	PM_2.5_	PM_10_
Final Conc. without plant C_0_ (ppm)	2.24	1.7	598	39	51
Final conc. with *Epipremnum aureum* Cf (ppm)	0.84	1.5	443	14	17
*Epipremnum aureum* RE%	62.5%	11.7%	25.91%	64.10%	66.66%
Final conc. with *Chlorophytum comosum* C_f_ (ppm)	0.51	1.4	453	6	6
*Chlorophytum comosum* RE%	77.23%	17.64%	24.24%	84.61%	88.23%
Final with *Syngonium* C_f_ (ppm)	0.41	0.5	411	0	0
*Syngonium* RE%	81.69%	70.58%	31.27%	100%	100%
Final conc. with *Cordyline fruticosa* Cf (ppm)	0.28	0.2	378	18	20
*Cordyline fruticosa* RE%	87.5%	88.23%	36.78%	53.84%	60.78%

## Discussion

### VOCs measurements in different field study

Our findings align with chambers studies by Tian et al.^20^, showing variability in removal efficiency from 39.96% to 100% for benzene. Sriprapat and Thiravetyan^21^ also reported high removal efficiency in species like *Epipremnum aureum* and *Chlorophytum comosum*. Notably, *Cordyline fruticosa* demonstrated the highest removal efficiency for benzene, toluene, and acetophenone (72%, 63.55%, and 74.55%). *Chlorophytum comosum*: Results matched studies by Suárez-Cáceres and Pérez-Urrestarazu^14^ showed that potted plants effectively reduced indoor air pollutants in sealed chambers removal efficiencies ranging from 10% to 90% for various VOCs. Regarding *Syngonium podophyllum* the results match NASA’s certification as an effective air purifier.

Further, our results support Bandehali et al.^1^ findings that potted plants can significantly reduce VOC concentrations in sealed chambers, with reductions ranging from 10% to 90% in 24 h. The removal efficiency of *Epipremnum aureum* for benzene matched the results from Treesubsuntorn and Thiravetyan^15^(56–59%). These findings are consistent with similar studies in the field, which have also highlighted the potential of *Cordyline* plants for indoor air quality improvement. The data analysis revealed that *Cordyline fruticosa* consistently demonstrates high efficiency in removing TVOCs across different study volume chambers and laboratory preparation room compared to *Epipremnum aureum*, *Chlorophytum comosum*, and *Syngonium podophyllum*.

Current research shows that *Cordyline fruticosa* from *Asparagaceae* family and *Epipremnum aureum* from *Araceae* family have higher removal per leaf area for TVOCs in all the three experimental set-ups of incubator chamber and laminar flow chamber and Lab. preparation room. These are agreed with Treesubsuntorn and Thiravetyan^15^ and Wannomai et al.^22^. In the case of a *Dracaena Sanderiana* under the light condition, higher benzene removal efficiency was noted, while *Scindapsus aureus* from the *areacea* family had the highest TMA removal efficiency Same results realized by Teiri et al.^18^ confidence the effectiveness of anther *Araceae* family species (*palm* species) for the removal of indoor formaldehyde by 65–100%^18^. Moreover, based on previous research, Su and Lin^23^ studied the efficacy of indoor plants like Cordyline, Schefflera, and Dodonaea for air purification particularly regarding the elimination of formaldehyde and carbon dioxide.

The removal efficiencies obtained in controlled environments (incubator and laminar flow chambers) were generally higher than those measured in the real pharmaceutical laboratory. This variation can be attributed to the more stable and enclosed nature of the chambers, which provided uniform VOC exposure and minimized air exchange. In contrast, the real-lab setting involved continuous air movement, variable chemical emissions, and natural ventilation, all of which influence pollutant dispersion and limit the contact time between VOCs and plant surfaces^23^. Despite these differences, the real-lab data reflect the true performance of plant-based biofilters under realistic operating conditions, confirming their practical potential for improving indoor air quality in occupied laboratory environments.

### IAQ parameters measurements

These results align with previous studies, such as Zhang et al.^3^, which found higher benzene removal rates for *E. aureum* compared to *C. comosum*. Comparative to other Studies related to Potted Plants in Virtually Sized Rooms like Wood et al.^25^ and Pettit et al.^26^ have shown significant VOC reduction using potted plants. Studies by Torpy et al.^27^ and Yau et al.^28^ showed that plants could control VOC concentrations between 20 and 300 parts per billion (ppb).

Our results are consistent with these findings, showing substantial VOC reduction in the laboratory environment. The CO_2_, CO, PM_2.5_, and PM_10_ levels were maintained within permissible limits after introducing the plants, confirming the efficiency of PBBFs in enhancing IAQ. These outcomes are corroborated by studies from Irga et al. (2018)^29^ and Ibrahim et al.^30^, which reported similar results using plant-based bio-filter systems or active green walls (AGWs).

Numerous investigations and research findings suggest the efficacy of *Cordyline fruticosa*, *Syngonium*,* Chlorophytum comosum*, and *Epipremnum aureum* in enhancing IAQ. Research by Han et al.^31^ has shown that *Cordyline fruticosa* is more effective than other plant species for eliminating organic laboratory volatile organic compounds (VOCs). In 2018, Torpy et al.^27^ examined the decline in the amounts of volatile organic compounds (VOCs) in different ketones and BTEX.

For the IAQ evaluation, the CO_2_, CO, and particulate matter (PM_2.5_, PM_10_) concentrations were all within allowable bounds. Even though the TVOC concentration for the organic laboratory was somewhat higher during the practical section and remained at the ASHRAE standard after installing the plant-based bio-filter (PBBF) represented in potted plants, the same result was reported by Yau et al.^28^. Active green walls (AGWs), which are composed of *Syngonium podophyllum*, *Chlorophytum comosum*, and *Epipremnum aureum*, are plant-based bio-filter systems that Irga et al.^29^ and Ibrahim et al.^30^ used to measure the quantity of volatile organic compounds (VOCs) eliminated by different plant species. The removal efficiencies of 54.5–6.04% for PM_2.5_, 65.42–9.27% for PM10, and 46–4.02% for VOCs were recorded using the BIAB system^30^. And this is supported by the conclusions drawn from our current investigation.

### Summary of findings

**Chamber studies**: With removal rates ranging from 10% to 90% in under 24 h, potting plants successfully reduced indoor air pollutants in sealed chambers. *Cordyline fruticosa* and *Epipremnum aureum* demonstrated the highest removal efficiencies for benzene and acetophenone.

**Field studies**: In the laboratory preparation room, *Epipremnum aureum* and *Syngonium podophyllum* performed better in reducing benzene and toluene. *Cordyline fruticosa* reduced acetophenone and benzaldehyde better than *Chlorophytum comosum*.

Recent studies help explain the systematic differences we observed between sealed-chamber and real-laboratory removal efficiencies. Chamber experiments and active botanical filters generate higher and more consistent single-pass removal because they provide controlled airflows, reduced dilution, and enhanced contact between polluted air and plant/soil compartments^24^ DOI:10.1016/j.buildenv.2025.112813). These mechanistic studies partition removal into leaf/stomatal uptake, surface adsorption, and rhizosphere/microbial degradation, showing that rhizosphere processes and medium composition become especially important in active or hydroponic systems. Conversely, in naturally ventilated, occupied laboratory rooms pollutant concentrations and air-exchange rates fluctuate continuously, limiting residence time and reducing plant pollutant contact; this dynamic behavior is also described and modeled in recent urban-climate and hybrid-system studies). Taken together, these findings indicate that higher chamber efficiencies do not contradict our field data but instead reflect differences in system boundary conditions (sealed vs. open), air mixing, and whether the system is passive (potted plants) or active (recirculating botanical filters). We have cited these recent works and used them to support our interpretation and our recommendation that future work should evaluate active recirculation and longer-duration exposures to quantify the maximum phytoremediation potential.

**Removal per leaf area and weight of VOCs taken by plants**: In every experimental situation, *Cordyline fruticosa* and *Epipremnum aureum* showed greater removal rates per leaf area for VOCs.

**IAQ parameters and VOC removal with indoor plants**: Among the plant species investigated, *Cordyline fruticosa* exhibits the lowest mean VOC level, measuring 0.36 ± 0.14. Nevertheless, the precise standards you employ to determine efficacy will determine whether it is the “most effective” in eliminating volatile organic compounds. When it came to PM_10_ and PM_2.5_ removal efficiency, *Syngonium podophyllum* outperformed *Epipremnum aureum*, *Chlorophytum comosum*, and *Cordyline fruticose*.

**Implementing applications**: Research studies and EPA estimates indicate the number of plants per unit area required to filter indoor air effectively; larger plants with higher leaf surface areas yield better results. In small areas like pharmaceutical labs, plant-based bio-filter indoor air purifiers offer a practical, cost-effective, and eco-friendly means of improving IAQ. Overall Impact on IAQ: By combining ventilation systems with plant-based bio-filters, significant improvements in IAQ can be attained, improving people’s health and productivity in indoor environments. These findings corroborate current research and highlight the potted plants’ potential as effective indoor air purifiers.

## Conclusion

This study underscores the effectiveness of phytoremediation technology using plant-based biofilters (PBBFs) to enhance indoor air quality (IAQ) by removing hazardous volatile organic compounds (VOCs). Among the tested species, Cordyline fruticosa exhibited the highest efficiency in eliminating benzene and acetophenone, while Epipremnum aureum demonstrated notable removal rates across multiple VOCs, particularly in controlled environments. Chlorophytum comosum and Syngonium podophyllum also showed strong VOC removal capabilities, with Syngonium excelling in toluene and acetophenone elimination. The findings suggest that a strategic combination of these plants can optimize air purification in pharmaceutical laboratories and other indoor settings. This study reinforces the potential of indoor plants as sustainable, natural air purifiers and provides a foundation for selecting effective species for targeted VOC mitigation. The most effective plant species for eliminating all contaminants examined was Cordyline fruticosa, which was particularly effective in getting rid of acetophenone and benzene. Syngonium podophyllum also showed strong effectiveness, especially in the removal of toluene and acetophenone. Both Epipremnum aureum and Chlorophytum comosum demonstrated efficacy, but with notable intercontaminant variation in their outcomes. The findings suggest that a carefully selected combination of these plants could significantly enhance indoor air quality by concentrating on a broad spectrum of VOCs.

The study results highlight PBBFs’ potential as an effective strategy to raise IAQ in interior environments, including pharmaceutical organic laboratories. The potential of indoor plants as natural air purifiers that can eliminate hazardous volatile organic compounds (VOCs) from indoor environments is supported by this study. Both *Cordyline fruticosa* and *Epipremnum aureum* showed excellent efficacy in eliminating distinct volatile organic compounds and serve as a foundation for selecting appropriate plants for targeted VOC mitigation.

### Limitations of the study

While the present study provides valuable insights into the phytoremediation potential of four indoor plant species under controlled conditions, several limitations should be acknowledged. The experiments were conducted over a short exposure period (40 min) and under static air conditions, which may not fully represent the plants’ long-term or dynamic performance in real indoor environments. In addition, the study employed potted plants rather than integrated or active bio-filter systems. Moreover, the VOC analysis was restricted to four representative compounds (benzene, toluene, benzaldehyde, and acetophenone) due to the detection range of the instrument used. Future research will aim to extend the exposure duration, incorporate recirculating biofiltration systems, and expand the VOC profiling to include a wider spectrum of indoor air pollutants, providing a more comprehensive understanding of long-term plant-based air purification performance.

## Recommendations

Integrate a variety of plant species to target a broad spectrum of VOCs. Position plants strategically maximize their exposure to polluted air. Also maintain plant health to ensure consistent VOC absorption rates. Furthermore, further studies should be conducted to investigate the long-term effects and optimal conditions for the use of different plant species such as PBBs. In addition to implementing the application of PBBFs, it considers environmental factors and plant maintenance such as light, temperature, humidity and other environmental conditions. Additionally, comparative studies measuring the performance of Cordyline compared to other air-purifying plants can provide significant insights for indoor air quality management techniques. Finally, self-awareness of increasing indoor quality in different indoor environments is increasing public knowledge of the benefits of indoor plants for air purification.

## Supplementary Information

Below is the link to the electronic supplementary material.

Supplementary Material 1

## Data Availability

The datasets generated and/or analysed during the current study are available from the corresponding author on reasonable request.
