# Supplementary material for: Comparative evaluation of air phytoremediation potential of four ornamental potted plants for ecofriendly biofilter applications
Source: Sci Rep. 2025 Dec 29;15:45659. doi: 10.1038/s41598-025-30145-8 (PMC12753763; doi:10.1038/s41598-025-30145-8)
Supplement: Supplementary file 1 — Supplementary Material 1 [file 41598_2025_30145_MOESM1_ESM.docx]

# Supplementary Material

Table S1. Leaf area measurements for each species used in the study. Leaf area was determined using ImageJ software (version 1.53e, NIH, USA) following a digital image-based method. Leaves were categorized into small, medium, and large sizes. Three representative leaves from each group were scanned to determine the average surface area (cm²). The total leaf area per plant was calculated using the following equation:

- A_total = Σ (A_avg,group × N_group)

Where A_total is the total leaf area per plant, A_avg,group is the average leaf area of each size group (cm²), and N_group is the number of leaves in that group. The same potted plants were used throughout all experimental trials; therefore, the number of leaves and total leaf area remained constant during the study period.

## Calculation Example

For example, for Epipremnum aureum:

A_total = (40.33 × 11) + (50.99 × 5) + (73.26 × 1)
A_total = 443.63 + 254.95 + 73.26 = 771.84 cm² ≈ 771.39 cm²

The same method was applied to all other species to determine their total leaf areas.

| Leaf category | Epipremnum aureum | Chlorophytum comosum | Syngonium podophyllum | Cordyline fruticosa |
| --- | --- | --- | --- | --- |
| Small leaf number | 11 | 0 | 10 | 3 |
| Small leaf area (cm²) | 40.33 ± 86.01 | 0 | 88.77 ± 85.16 | 45.57 ± 86.91 |
| Medium leaf number | 5 | 12 | 3 | 7 |
| Medium leaf area (cm²) | 50.99 ± 85.04 | 32.47 ± 85.16 | 93.57 ± 88.07 | 56.99 ± 85.03 |
| Large leaf number | 1 | 30 | 3 | 5 |
| Large leaf area (cm²) | 73.26 ± 85.84 | 36.07 ± 89.61 | 95.04 ± 90.35 | 62.78 ± 87.10 |
| Total leaf area (cm²) | 771.39 | 1471.77 | 1453.57 | 849.56 |

Note: The total leaf area (A_total) for each species was derived using the above formula. Leaf number and area were consistent across all experimental trials.
